# Orchid diseases caused by *Fusarium oxysporum* species complex in Taiwan

**DOI:** 10.3389/fpls.2025.1630094

**Published:** 2026-01-19

**Authors:** An Chang, Che-Wei Chang, Cheng-Chun Wu, Kuo-Hsi Lin, Nittaya Chookoh, Jintana Unartngam, Wen-Hsin Chung

**Affiliations:** 1 Department of Plant Pathology, National Chung Hsing University, Taichung, Taiwan; 2 Tungs’ Taichung MetroHarbor Hospital, Taichung, Taiwan; 3 Department of Post-Baccalaureate Medicine, National Chung Hsing University, Taichung, Taiwan; 4 Department of Horticulture, Faculty of Agriculture, Kasetsart University, Bangkok, Thailand; 5 Department of Plant Pathology, Faculty of Agriculture at Kamphaeng Saen, Kasetsart University, Nakhon Pathom, Thailand; 6 Master Program for Plant Medicine and Good Agricultural Practice, National Chung Hsing University, Taichung, Taiwan

**Keywords:** orchid, *Fusarium oxysporum* species complex, taxonomy, morphology, MLST (multilocus sequence typing)

## Abstract

Orchid diseases caused by *Fusarium* spp. are common in orchid gardens worldwide, with *F. oxysporum* being the most dominant species. *F. oxysporum* is defined as a species complex, FOSC. In Taiwan, orchids are highly diverse, and certain species are economically important. However, orchid diseases caused by FOSC remain unclear. In this study, 63 FOSC isolates were collected from commercial orchids, including five epiphytic, one semi-terrestrial, and two terrestrial orchids. Terrestrial orchids were the major hosts of isolated FOSC (41/63). The isolates were confirmed to be pathogenic through mycelium plug or spore suspension inoculation, and they were subsequently used for further analyses. Phylogenetic analyses indicated that FOSC isolates could be separated into six taxa, *F. contaminatum*, *F. cugenangense*, *F. curvatum*, *F. nirenbergiae*, *F. odoratissimum*, and *F. triseptatum*, based on *cmdA*, *rpb2*, *tef1*, and *tub2* gene sequences. This classification is also associated with morphological characteristics. These results provide a preliminary insight into pathogenic FOSC in orchids and can be used to explore potential resistant cultivars or screen for effective management agents.

## 1 Introduction

Orchids are monocotyledonous plants belonging to the family Orchidaceae. This family constitutes 10% of the flowering plants, with over 899 genera and 27,800 species worldwide (Haider et al., 2012; Givnish et al., 2016). Among the orchid species, approximately 73% are epiphytes. Orchids are known to thrive in warm and humid environments, particularly in tropical and subtropical areas (Haider et al., 2012; Smitamana and McGovern, 2018). Taiwan is located in a subtropical region with a warm and humid climate that contributes to an abundance of orchid species, including 97 genera and 477 native species in Taiwan (Lin, 2024). This provides a rich genetic resource for highly diverse artificial hybrid breeding. As a result, the orchid industry is well-developed in Taiwan (Hsiao et al., 2011). According to the Bureau of Foreign Trade, Ministry of Economic Affairs in Taiwan and the Taiwan Orchid Growers Association, the export value of live orchids (including tissue culture seedlings and cut flowers) in 2024 was approximately 190 million USD, with the top species being *Phalaenopsis* followed by *Oncidium*, *Cymbidium*, *Dendrobium*, *Cattleya*, and *Paphiopedilum* (https://portal.sw.nat.gov.tw/APGA/GA30; https://www.togacloud.org.tw/).

Orchid diseases caused by *Fusarium* spp. are prevalent worldwide. Over eight *Fusarium* species are known to infect and cause diseases in orchids (Srivastava et al., 2018). The entire orchid plant can be infected with *Fusarium* pathogens, including the roots, stems, leaves, and flowers. Young seedlings and shoots can easily exhibit symptoms during the growth period (Swett and Uchida, 2015). Thus, these *Fusarium* pathogens can cause seedling death under favorable environmental conditions (Srivastava et al., 2018). Among these *Fusarium* spp., *F. oxysporum* species complex (FOSC) are common pathogens that cause diseases in orchids (Srivastava et al., 2018). FOSC has been reported to infect most orchids, including *Cattleya*, *Cymbidium*, *Dendrobium*, *Paphiopedilum, Phalaenopsis*, and *Vanilla planifolia*, and has been recorded in many countries (Swett and Uchida, 2015; Srivastava et al., 2018). In addition to these hosts, FOSC isolates can cause diseases in *Anoectochilus formosanus*, *Vanda*, and *Oncidium* (Simmonds, 1966; Alfieri, 1984; Huang et al., 2014; Srivastava et al., 2018).

The primary classification of FOSC in the early stages is based on morphological characteristics and host specificity (Snyder and Hansen, 1940; 1941). According to host specificity, *formae* sp*eciales* are defined. Over 106 *formae* sp*eciales* (ff. spp.) of FOSC (Edel-Hermann and Lecomte, 2019) were recorded because of their host specificity. However, conducting disease assays to classify each *formae* sp*eciales* is both time-consuming, laborious and expensive sp. To complete traditional host specificity tests, pathogenicity assays must include multiple host species. The progress is time-consuming, not only because of the need to collect and maintain host plants, but also due to the waiting for symptom development. A molecular categorization method using Multilocus Sequence Typing (MLST) is more convenient, offering immediate updates and unified definitions. However, the potentially higher costs should be considered. Currently, FOSC are classified into eight clades based on β-tubulin II (*tub2*), calmodulin (*cmdA*), the second largest subunit of DNA-dependent RNA polymerase II (*rpb2*), and translation elongation factor (*tef1*) gene sequences (Lombard et al., 2019). This method provides a clear and easy-to-use reference.

Although isolates causing orchid disease have been recorded in several articles, molecular information is limited in the database. In the early years, only isolates obtained from *A. formosanus* (Huang et al., 2014), *Cattleya* (Pedroso-de-Moraes et al., 2011), *Dendrobium* (Xiao et al., 2012), and *V. planifolia* (Pinaria et al., 2015) had the rDNA intergenic spacer region, rDNA internal transcribed spacer (ITS) region, mitochondrial small subunit ribosomal RNA (*mtSSU*) gene, or *tef1* gene sequence data individually, but in limited quantities. Additional sequence data related to isolates from *Cymbidium* (Jin-Ai et al., 2018; Huang et al., 2020), *Dendrobium* (Zhang et al., 2017; Sarsaiya et al., 2020; Xiao and Li, 2021), and *V. planifolia* (Flores-de la Rosa et al., 2018) have been published in subsequent years. Recently, additional MLST studies have been conducted on the FOSC pathogens in orchids. Mirghasempour et al. (2022) employed the system developed by Lombard et al. (2019), whereas Yang J. et al. (2024) used the MLST method with the ITS region, the largest subunit of the DNA-dependent RNA polymerase I (*rpb1*), *rpb2*, and *tef1* gene sequences, to classify FOSC isolates from *Dendrobium*. Nevertheless, further supplementation of the sequence data associated with orchid-pathogenic FOSC isolates is required.

In Taiwan, FOSC causes yield losses in several crops. *Fusarium* wilt commonly occurs in cucurbits, such as melon (Chang et al., 2024; Chai et al., 2025) and luffa (Namisy et al., 2023). Moreover, FOSC infects vegetables and ornamentals, such as wilting and yellows on Brassicaceae crops (Lin et al., 2014; Chu et al., 2024), and wilting on lisianthus (Wu et al., 2023). Likewise, diseases caused by *Fusarium* spp. including FOSC in orchids are common in growing facilities. In previous studies, FOSC have been reported to cause leaf blight, stem rot, and root rot in orchids, including *A. formosanus, Cymbidium*, *Paphiopedilum*, and *Phalaenopsis* (Su et al., 2012; Huang et al., 2014; Tzean, 2019). However, information on FOSC in Taiwanese orchids remains obscure. Based on information published by Srivastava et al. (2018), FOSC can infect and cause disease in seven types of orchids. Most studies have focused on individual orchid species, leaving a gap in comprehensive information regarding FOSC species across different orchids in Taiwan. In this study, we focused on orchids that occupy a large proportion of the market, including *Cattleya*, *Cymbidium*, *Dendrobium*, *Oncidium*, *Paphiopedilum*, and *Phalaenopsis*. This study aimed to 1) investigate orchid diseases in commercial plants, including those in epiphytic, terrestrial, and semi-terrestrial orchids, caused by FOSC; 2) classify the pathogen species of FOSC from orchids based on phylogenetic analyses providing more sequence data about these pathogens; and 3) determine their morphological characteristics.

## 2 Materials and methods

### 2.1 Investigation and fungal collection of FOSC

From 2018 to 2021, Orchidaceae plants, including *Calanthe* sp*eciose* (*Cas*), *Cattleya* (*Cat*), *Chysis limminghis* (*Ch*), *Cymbidium* (*Cy*), *Dendrobium* (*De*), *Epidendrum* (*Ep*), *Haraella retrocalla* (*Ha*), *Maxillaria* (*Ma*), *Oncidium* (*On*), *Paphiopedilum* (*Pa*), *Phalaenopsis* (*Ph*), *Renanthera* (*Re*), *Tuberolabium kotoense* (*Tu*), *Vanda* (*Va*), and *Vanilla planifolia* (*Vap*) showing rot or blight in roots, stems, leaves, or shoots, were collected from orchid gardens located in Changhua, Chiayi, Nantou, Pingtung, Taichung, Tainan, Taoyuan, and Yunlin in Taiwan. Eight locations were visited, with 33 investigations. Pieces of diseased tissues (5 × 5 mm) were cut and sterilized with 1% NaClO for 30 s, followed by rinsing three times with sterilized distilled water, dried using sterilized paper, and cultured on water agar (2% WA; Fei Kung Agar-Agar, Tainan, Taiwan) at 28°C with 12-h light daily for 1–3 d. Then, a single mycelium was cut and cultured on potato dextrose agar plates (PDA; BD Difco™, New Jersey, USA) at 28 °C with 12-h light daily for 5–7 d. The morphologies of colonies and conidia were observed according to the methods described by Leslie and Summerell (2006). FOSC-like isolates were cultured in pure culture medium using single spores. The purified isolates were cultured on PDA at 28°C with 12-h light daily for 7 d; then, two 2–3 mm^2^ agar plugs from the colony margins were transferred to 5-mL glass store tubes (with 10% sand soil and 1% agar) and maintained for long-term storage.

### 2.2 Pathogenicity test

The FOSC isolates used in this study satisfied Koch’s postulates. The plants used in pathogenicity test were recorded in **Table 1**. Most isolates were inoculated on the same species from which they were originally obtained. However, some isolates were tested on different species because of limitations in price or availability. The orchids used for inoculation were 1–2 years old plants. Some of them inoculated with tissue culture seedlings were 4–6 months, such as *Ph*. The FOSC isolates were grown on PDA plates for 7–14 d at 28°C with a 12-h light period per day. The orchid plants were sterilized using a paper towel with 75% ethanol, and wounds were created using a needle (1–5 mm depth) on the leaves, stems, pseudobulbs, or crowns of the orchid plants. Inoculation was conducted by two methods, mycelium plug inoculation and spore suspension inoculation. The mycelium inoculation method was adapted from Chung et al. (2011). Three mycelium plugs were placed on each wound site, and PDA agar plugs were used as the control treatment. The spore suspension inoculation method was adapted from Huang et al. (2014). The spore suspension (1 × 10^7^ spores/mL) was prepared by washing spores with sterilized ddH_2_O, followed by filtration through Miracloth. The 10–20 μL spore suspension mixed 1:1 (v/v) with 0.2% WA was dropped on each of the four wounds, and sterilized ddH_2_O was used as negative control. The inoculation methods for each isolate were shown in **Table 1**. Inoculated plants were incubated in a growth chamber at 28°C under a 12-h light period per day, and symptoms were recorded 7–14 d after inoculation, depending on the orchid species.

**Table 1:** The 63 *Fusarium oxysporum* species complex isolates obtained from orchid hosts were used to test pathogenicity in this study.

Host	Isolate code	Source	Tissue	Origin	Longitude and latitude^a^	Collection date	Plants used in pathogenicity test^b^	Inoculation method^c^	Pathogenicity^d^
*Cattleya*	Ca5	*Cattleya* sp.	Pseudostem	Mingjian Township, Nantou County	23.862, 120.679	2021/07/16	same as isolation source	S	+
*Cymbidium*	Cy25	*Cybidium sinense*	Pseudobulb	Houli District, Taichung City	24.329, 120.723	2018/08/15	same as isolation source	S	+
	Cy28	*Cy. sinense*	Pseudobulb	Houli District, Taichung City	24.329, 120.723	2018/08/15	same as isolation source	S	+
	Cy32	*Cy. sinense*	Pseudobulb	Houli District, Taichung City	24.329, 120.723	2018/08/15	same as isolation source	S	+
	Cy41	*Cy. sinense*	Pseudobulb	Houli District, Taichung City	24.329, 120.723	2018/08/21	same as isolation source	S	+
	CyB04	*Cy. ensifolium*	Pseudobulb	South District, Taichung City	24.12, 120.67	2019/04/23	same as isolation source	S	+
	CyB05	*Cy. ensifolium*	Pseudobulb	South District, Taichung City	24.12, 120.67	2019/04/23	same as isolation source	S	+
	CyB06	*Cy. ensifolium*	Pseudobulb	South District, Taichung City	24.12, 120.67	2019/04/23	same as isolation source	S	+
	CyB07	*Cy. ensifolium*	Pseudobulb	South District, Taichung City	24.12, 120.67	2019/04/23	same as isolation source	S	+
	CyB13	*Cy. ensifolium*	Pseudobulb	South District, Taichung City	24.12, 120.67	2019/04/23	same as isolation source	S	+
	CyB14	*Cy. ensifolium*	Pseudobulb	Houli District, Taichung City	24.329, 120.723	2019/03/21	same as isolation source	S	+
	CyB24	*Cy. ensifolium*	Pseudobulb	Yuchi Township, Nantou County	23.887, 120.916	2020/02/03	same as isolation source	S	+
	CyB26	*Cy. ensifolium*	Pseudobulb	Yuchi Township, Nantou County	23.887, 120.916	2020/02/03	same as isolation source	S	+
	CyB27	*Cy. ensifolium*	Pseudobulb	Yuchi Township, Nantou County	23.887, 120.916	2020/02/03	same as isolation source	S	+
	CyB30	*Cy. ensifolium*	Pseudobulb	Yuchi Township, Nantou County	23.887, 120.916	2020/02/03	same as isolation source	S	+
	CyB31	*Cy. ensifolium*	Pseudobulb	Yuchi Township, Nantou County	23.887, 120.916	2020/02/03	same as isolation source	S	+
	CyB33	*Cy. ensifolium*	Pseudobulb	Yuchi Township, Nantou County	23.887, 120.916	2020/02/03	same as isolation source	S	+
	CyB34	*Cy. ensifolium*	Pseudobulb	Yuchi Township, Nantou County	23.887, 120.916	2020/02/03	same as isolation source	S	+
	CyB35	*Cy. ensifolium*	Pseudobulb	Yuchi Township, Nantou County	23.887, 120.916	2020/02/03	same as isolation source	S	+
	CyB41	*Cy. ensifolium*	Pseudobulb	Yuchi Township, Nantou County	23.887, 120.916	2020/02/03	same as isolation source	S	+
	CyB43	*Cy. ensifolium*	Pseudobulb	Meishan Township, Chiayi County	23.583, 120.567	2020/02/13	same as isolation source	S	+
	CyB55	*Cy. ensifolium*	Pseudobulb	Gukeng Township, Yunlin County	23.593, 120.569	2020/02/13	same as isolation source	S	+
	CyB57	*Cy. ensifolium*	Pseudobulb	Gukeng Township, Yunlin County	23.593, 120.569	2020/02/13	same as isolation source	S	+
	CyB58	*Cy. ensifolium*	Pseudobulb	Gukeng Township, Yunlin County	23.593, 120.569	2020/02/13	same as isolation source	S	+
	CyB61	*Cy. ensifolium*	Pseudobulb	Meishan Township, Chiayi County	23.583, 120.567	2020/02/13	same as isolation source	S	+
	CyB63	*Cy. ensifolium*	Pseudobulb	Meishan Township, Chiayi County	23.583, 120.567	2020/02/13	same as isolation source	S	+
	CyB65	*Cy. ensifolium*	Pseudobulb	Meishan Township, Chiayi County	23.583, 120.567	2020/02/13	same as isolation source	S	+
	CyB67	*Cy. ensifolium*	Pseudobulb	Meishan Township, Chiayi County	23.583, 120.567	2020/02/13	same as isolation source	S	+
*Dendrobium*	De18	*Dendrobium nobile* hybrid	Pseudostem	Mingjian Township, Nantou County	23.843, 120.683	2021/07/29	same as isolation source	S	+
	De23	*D. nobile* hybrid	Pseudostem	Mingjian Township, Nantou County	23.843, 120.683	2021/07/29	same as isolation source	S	+
	De24	*D. nobile* hybrid	Pseudostem	Mingjian Township, Nantou County	23.843, 120.683	2021/07/29	same as isolation source	S	+
	De29	*Dendrobium* sp.	Pseudostem	Douliu City, Yunlin County	23.724, 120.558	2021/09/08	*D. nobile* hybrid	M	+
	De30	*Dendrobium* sp.	Pseudostem	Douliu City, Yunlin County	23.724, 120.558	2021/09/08	*D. nobile* hybrid	S	+
*Haraella retrocalla*	Ha1-1	*Haraella retrocalla*	Stem	South District, Taichung City	24.103, 120.681	2022/05/12	same as isolation source	M	+
*Maxillaria tenuifolia*	Ma6	*Maxillaria tenuifolia*	Pseudobulb	Houli District, Taichung City	24.329, 120.723	2020/10/15	same as isolation source	M	+
*Paphiopedilum*	Pa17	*Paphiopedilum* sp.	Leaf	Dacun Township, Changhua County	23.993, 120.560	2021/07/15	*Pa. callosum*	M	+
	Pa18	*Paphiopedilum* sp.	Leaf	Puli Township, Nantou County	23.973, 121.005	2021/08/16	*Pa. callosum*	M	+
	Pa19	*Paphiopedilum* sp.	Leaf	Puli Township, Nantou County	23.973, 121.005	2021/08/16	*Pa. callosum*	M	+
	Pa23	*Pa. callosum*	Leaf	Puli Township, Nantou County	23.973, 121.005	2021/08/16	same as isolation source	M	+
	Pa24	*Pa. callosum*	Leaf	Puli Township, Nantou County	23.973, 121.005	2021/08/16	same as isolation source	S	+
	Pa25	*Pa. callosum*	Leaf	Puli Township, Nantou County	23.973, 121.005	2021/08/16	same as isolation source	M	+
	Pa26	*Pa. callosum*	Leaf	Puli Township, Nantou County	23.973, 121.005	2021/08/16	same as isolation source	M	+
	Pa27	*Pa. callosum*	Leaf	Puli Township, Nantou County	23.973, 121.005	2021/08/16	same as isolation source	M	+
	Pa28	*Pa. callosum*	Leaf	Puli Township, Nantou County	23.973, 121.005	2021/08/16	same as isolation source	M	+
	Pa29	*Pa. callosum*	Leaf	Puli Township, Nantou County	23.973, 121.005	2021/08/16	same as isolation source	M	+
*Vanda*	Va3	*Vanda ampullaceum*	Leaf	Huwei Township, Yunlin County	23.715, 120.422	2021/09/08	*Vanda lamellata*	M	+
	Va40-2	*Arachnis* x *Vanda*	Leaf	Zhutian Township, Pingtung County	22.595, 120.532	2021/11/12	*Vanda lamellata*	M	+
	Va45	*Arachnis* x *Vanda*	Leaf	Zhutian Township, Pingtung County	22.595, 120.532	2021/11/12	*Vanda lamellata*	M	+
	Va48	*Arachnis* x *Vanda*	Leaf	Zhutian Township, Pingtung County	22.595, 120.532	2021/11/12	*Vanda lamellata*	M	+
*Vanilla planifolia*	VaP1	*Vanilla planifolia*	Stem	Puli Township, Nantou County	23.969, 120.990	2021/10/13	same as isolation source	M	+
	VaP2-1	*Vani. planifolia*	Stem	Puli Township, Nantou County	23.969, 120.990	2021/10/13	same as isolation source	M	+
	VaP3-1	*Vani. planifolia*	Stem	Puli Township, Nantou County	23.969, 120.990	2021/10/13	same as isolation source	M	+
	VaP4	*Vani. planifolia*	Stem	Puli Township, Nantou County	23.969, 120.990	2021/10/13	same as isolation source	M	+
	VaP5	*Vani. planifolia*	Stem	Puli Township, Nantou County	23.969, 120.990	2021/10/13	same as isolation source	M	+
	VaP6	*Vani. planifolia*	Stem	Puli Township, Nantou County	23.969, 120.990	2021/10/13	same as isolation source	M	+
	VaP12	*Vani. planifolia*	Stem	Puli Township, Nantou County	23.969, 120.990	2021/11/08	same as isolation source	M	+
	VaP14	*Vani. planifolia*	Stem	Puli Township, Nantou County	23.969, 120.990	2021/11/08	same as isolation source	M	+
	VaP15	*Vani. planifolia*	Stem	Puli Township, Nantou County	23.969, 120.990	2021/11/08	same as isolation source	M	+
	VaP17-1	*Vani. planifolia*	Stem	Puli Township, Nantou County	23.969, 120.990	2021/11/08	same as isolation source	M	+
	VaP20	*Vani. planifolia*	Stem	Puli Township, Nantou County	23.969, 120.990	2021/11/08	same as isolation source	M	+
	VaP22	*Vani. planifolia*	Stem	Puli Township, Nantou County	23.969, 120.990	2021/11/08	same as isolation source	M	+
	VaP23	*Vani. planifolia*	Stem	Puli Township, Nantou County	23.969, 120.990	2021/11/08	same as isolation source	M	+
	VaP24	*Vani. planifolia*	Stem	Puli Township, Nantou County	23.969, 120.990	2021/11/08	same as isolation source	M	+

a Geographic coordinates indicate approximate locations of sampling sites.

b List the plants used in pathogenicity test. “same as isolation source”: pathogenicity test conducted on the same host species from which the isolate was originally obtained.

c M: inoculation conducted by mycelium plug. S: inoculation conducted by spore suspension.

d +: isolates completed Koch’s postulates and their pathogenicity was confirmed.

### 2.3 Total DNA extraction

DNA extraction was performed as described by Saitoh et al. (2006) with some modifications. After culturing on PDA at 28°C with 12-h light daily for 10 d, the mycelia of isolates were scraped and transferred into 1.5-mL microcentrifuge tubes with 500 μL of a lysis solution (200 mM Tris-HCl, 50 mM ethylenediaminetetraacetic acid, 200 mM NaCl, and 1% n-lauroylsarcosine sodium salt at pH 8.0) and placed at −20°C for 24 h. Once lysis was completed, 500 μL of phenol:chloroform:isoamyl alcohol (25:24:1) was added to the tubes and mixed gently. The mixture was then centrifuged at 16,200 × *g* for 10 min. Next, the supernatant was collected in a new microcentrifuge tube with 240 μL of isopropanol and placed at −20°C for 1 h. To obtain the DNA pellet, the mixture was centrifuged at 16,200 × *g* for 5 min, and the supernatant removed, followed by the addition of 700 μL of 70% ethanol at −20°C, centrifuged at 16,200 × *g* for 1 min, and the supernatant removed. After placing the DNA pellet in a laminar flow hood to dry, 30 μL milli-Q water was added, and the mixture was placed at 5°C in an incubator for 15 min. The DNA samples were stored at −20°C for subsequent analysis.

### 2.4 Polymerase chain reaction amplification and sequencing

The primer pairs and PCR program used in this study are listed in **Supplementary Table S1**. For FOSC amplification, *cmdA*, *rpb2*, *tef1*, and *tub2* were used. Primers Cal228F/CAL2Rd were used for *cmdA* (Carbone and Kohn, 1999), RPB2-F/RPB2-R for *rpb2* (Wu et al., 2023), EF1/EF2 for *tef1* (O’Donnell et al., 1998), and T1/CYLTUB1R for *tub2* (O'Donnell and Cigelnik, 1997; Crous et al., 2004). According to Lombard et al. (2019), *tef1* provides the highest resolution among these gene regions. Therefore, *tef1* was selected for preliminary identification of isolates. The PCR mixtures contained 5 μL of PCR Master mix II (Genemark Technology Co., Ltd, Taiwan), 0.5 μL of each 10 mM primer, 18 μL of milli-Q water, and 1 μL of the DNA sample. All PCR products were subjected to electrophoresis analysis on a 1% TAE (40 mM Tris, 20 mM sodium acetate, and 1 mM ethylenediaminetetraacetic acid, pH 7.5) agarose gel to check their size and were sent to Tri-I Biotechnology Co., Ltd., Taiwan for purification and sequencing. To ensure sequence accuracy, PCR products were subjected to bidirectional sequencing and were assembled using BioEdit version 7.0.5.3 (Hall, 1999). Chromatograms were carefully reviewed to confirm base calling. The sequences were uploaded to National Center for Biotechnology Information (NCBI) via DDBJ.

### 2.5 Multilocus phylogenetic analyses

The assembled DNA fragments were aligned with reference sequences (**Supplementary Table S2**) from the NCBI database using MEGA version 7.0.26 (Kumar et al., 2016) with ClustalW. The aligned sequence fragments of each gene or region were merged for multilocus phylogenetic analyses. Four loci, *cmdA*, *rpb2*, *tef1*, and *tub2*, were analyzed. The merged sequences were subjected to Maximum Likelihood analysis to identify related taxa. To confirm the best evolutionary model, JModelTest version 2.0 (Posada, 2008) was used. Maximum likelihood analyses were performed with RAxML-ng v1.2.2 (Kozlov et al., 2019) using 1000 bootstrap replicates. MrBayes v.3.2.6 (Ronquist and Huelsenbeck, 2003) was used to construct a Bayesian inference tree. A Markov Chain Monte Carlo algorithm was used to calculate the random tree topology, which lasted for at least 4.5 M generations. A Markov Chain Monte Carlo analysis was performed until the average standard deviation of the split frequencies was below 0.01 with trees saved every 100 generations. The first 1,000 trees were discarded, and the remaining trees were used to determine the posterior probabilities.

### 2.6 Morphological characteristics of FOSC species

Single conidia of FOSC isolates were cultured on PDA and incubated at 28°C with 12-h light daily for 7 d in the incubator. PDA was used to record colonies and colonial pigments. Carnation leaf-piece agar (CLA) was used to observe FOSC conidial characteristics (Fisher et al., 1982). FOSC isolates were cultured on CLA plates and incubated at 24°C with 12-h blue light daily for 10 d. Conidial characteristics included the shape and size of the microconidia and macroconidia. Spezieller Nährstoffarmer agar was prepared to observe the chlamydospores. The conidial morphology of the isolates was observed using a Zeiss EL-Einsatz Axiophot 156 microscope (Carl Zeiss, Jena, Germany), and images were recorded using a Zeiss Axiocam 105 color camera (Carl Zeiss).

## 3 Results

### 3.1 Field investigation and fungal isolation of FOSC

**Figure 1:**
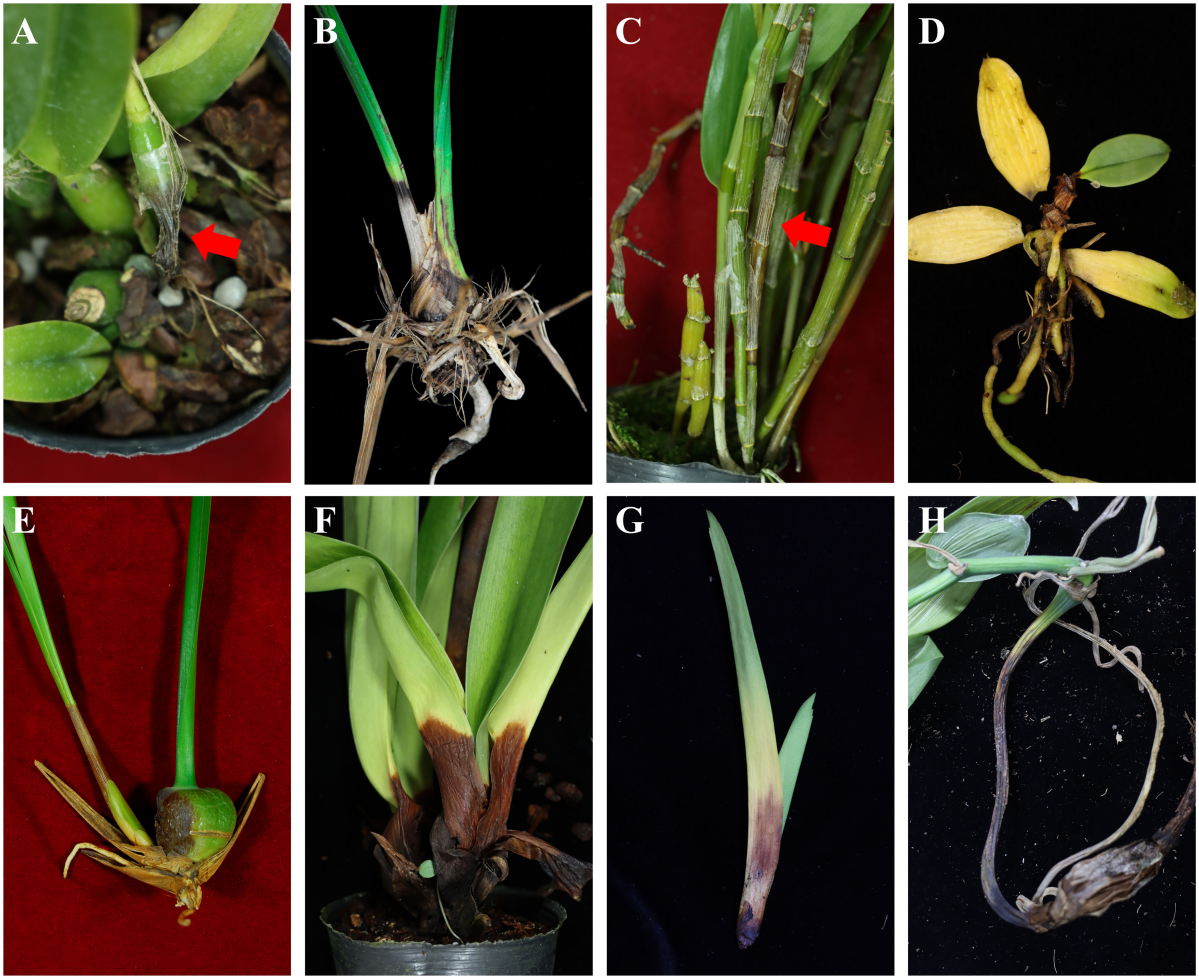
Orchids collected in this study showing symptoms caused by *Fusarium oxysporum* species complex (FOSC) infection. **(A)***Cattleya* showing pseudostem rot; **(B)***Cymbidium* showing pseudobulb; **(C)***Dendrobium* showing pseudostem rot; **(D)***Haraella retrocalla* showing stem rot; **(E)***Maxillaria* showing pseudobulb rot; **(F)***Paphiopedilum* showing basal leaf dry rot; **(G)***Vanda* showing basal leaf rot; **(H)***Vanilla planifolia* showing basal stem rot.

The orchids collected in this study are listed in **Table 2**, including 63 FOSC isolates. Most were collected from cultivation facilities, whereas others were collected from flower markets throughout Taiwan (**Table 1**). The source, origin, longitude and latitude, and collection date were recorded in **Table 1**. Eight major orchids, including *Cat*, *Cy, De*, *Ha*, *Ma*, *Pa*, *Va*, and *Vap*, were collected (**Figure 1**). Among the diseased plants, stem/pseudostem/pseudobulb rot was the predominant symptom in most orchids, and lesions on the leaves were minor (**Table 2**, **Figure 1**). The most common symptoms were pseudobulb rot in *Cy*, basal leaf dry rot in *Pa*, and stem rot in *Vap* (**Table 2**, **Figures 1B, F, H**). In the case of pseudobulb rot in *Cy*, leaf yellowing may show at the initial stage, then the entire pseudobulb might become completely rotted with foul smell develops (**Figure 1B**); meanwhile, the diseased *Cy* dies latterly. Here, we found the pathogens might remain latent until conditions become favorable for disease development. For stem rot in *De*, certain rot symptoms involve yellowing of the entire stem. In the case of basal leaf dry rot or leaf blight in *Pa*, the rot begins at the leaf base and gradually expand to approximately half of the leaf, ultimately causing symptomatic leaves to detach from the plant (**Figure 1F**). For stem and basal leaf rot in *Va*, rot initially occurs in the stem, and as the disease progresses, pronounced symptoms develop at the bases of young leaves, accompanied by yellowing (**Figure 1G**). In the case of *Vap*, leaves, stems, roots and beans have the potential to be infected. The roots show rot with yellowing. They also have latent infections in *Vap*, which may become symptomatic when *Vap* is unhealthy or lacks water. Finally, the diseased section of the stem dies off (**Figure 1H**). Based on the *tef1* sequence, these *Fusarium*-like isolates were confirmed as FOSC. The results showed that FOSC were the dominant pathogens causing diseases in terrestrial orchids (*Cy* and *Vap*). Fewer FOSC isolates were obtained from epiphytic orchids. Only a few FOSC isolates were obtained from *Cat*, *Ha*, and *Ma.* Therefore, these three orchids may not be major hosts of FOSC in Taiwan.

**Table 2:** Diseased orchids collected and number of isolates of *Fusarium oxysporum* species complex (FOSC) from different orchid hosts.

Growth forms	Host	Host abbreviation	Symptom	Number of isolation^a^
Terrestrial	*Calanthe* sp*eciose*	*Cas*	Pseudobulb rot	0
	*Cymbidium*	*Cy*	Pseudobulb/shooting rot	27
	*Vanilla planifolia*	*Vap*	Basal stem rot	14
Semi-terrestrial	*Paphiopedilum*	*Pa*	Basal leaf dry rot; leaf lesion	10
Epiphytic	*Cattleya*	*Cat*	Pseudostem rot; leaf lesion	1
	*Chysis limminghis*	*Ch*	Fleck spot	0
	*Dendrobium*	*De*	Pseudostem rot; leaf lesion	5
	*Epidendrum*	*Ep*	Pseudostem rot	0
	*Haraella retrocalla*	*Ha*	Stem rot	1
	*Maxillaria*	*Ma*	Pseudobulb rot	1
	*Oncidium*	*On*	Pseudobulb rot/petal drop	0
	*Phalaenopsis*	*Ph*	Leaf necrosis	0
	*Renanthera*	*Re*	Petal rot	0
	*Tuberolabium kotoense*	*Tu*	Basal leaf rot	0
	*Vanda*	*Va*	Stem/basal leaf rot	4

a Species identification was based on morphology and partial translation elongation factor (*tef1α*) sequence. The isolates were subjected to Koch’s postulates, and their pathogenicity was successfully confirmed.

### 3.2 Pathogenicity tests

**Figure 2:**
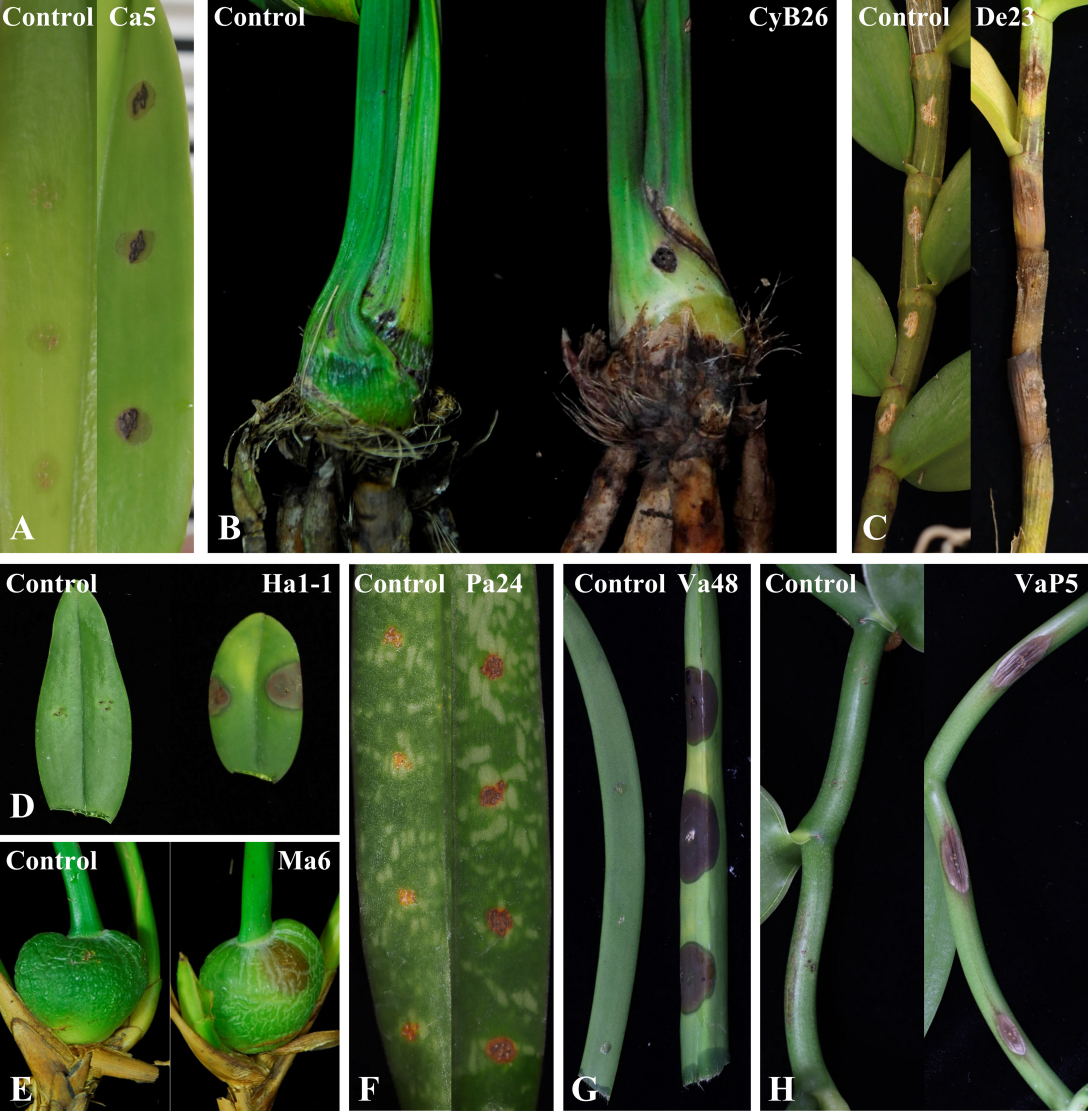
Pathogenicity results of *Fusarium oxysporum* species complex (FOSC) isolates from orchids. Control (left) and inoculated tissue (right) are shown in each panel. Control for the spore suspension method: sterilized water mixed 1:1 (v/v) with 0.2% WA; control for the mycelium plug method: PDA agar plug. The day-post inoculation (DPI) and inoculation methods were provided for each panel. **(A)** Isolate Ca5 from *Cattleya* (7 DPI; spore suspension); **(B)** Isolate CyB26 from *Cymbidium* (10 DPI; spore suspension); **(C)** Isolate De23 from *Dendrobium* (14 DPI; spore suspension); **(D)** Isolate Ha1–1 from *Haraella retrocalla* (7 DPI; mycelium plug); **(E)** Isolate Ma6 from *Maxillaria* (7 DPI; mycelium plug); **(F)** Isolate Pa24 from *Paphiopedilum* (14 DPI; spore suspension); **(G)** Isolate Va48 from *Vanda* (7 DPI; mycelium plug); **(H)** Isolate VaP5 from *Vanilla planifolia* (7 DPI; mycelium plug).

In this study, 63 FOSC isolates were examined and their pathogenicity was confirmed using Koch’s postulates (**Tables 1**, **2**). The pathogenicity test showed that all isolates could infect the original hosts or alternative testing hosts (some isolates from *De*, *Pa*, and *Va*), resulting in similar symptoms on the leaves, basal leaves, pseudostems, basal stems, pseudobulbs, or (**Table 1**). **Figure 2** displayed symptoms after inoculation, with one representative image shown for each orchid. Day post-inoculation and inoculation methods for each isolate were specified in the figure legends.

### 3.3 Multilocus phylogenetic analyses in FOSC

**Figure 3:**
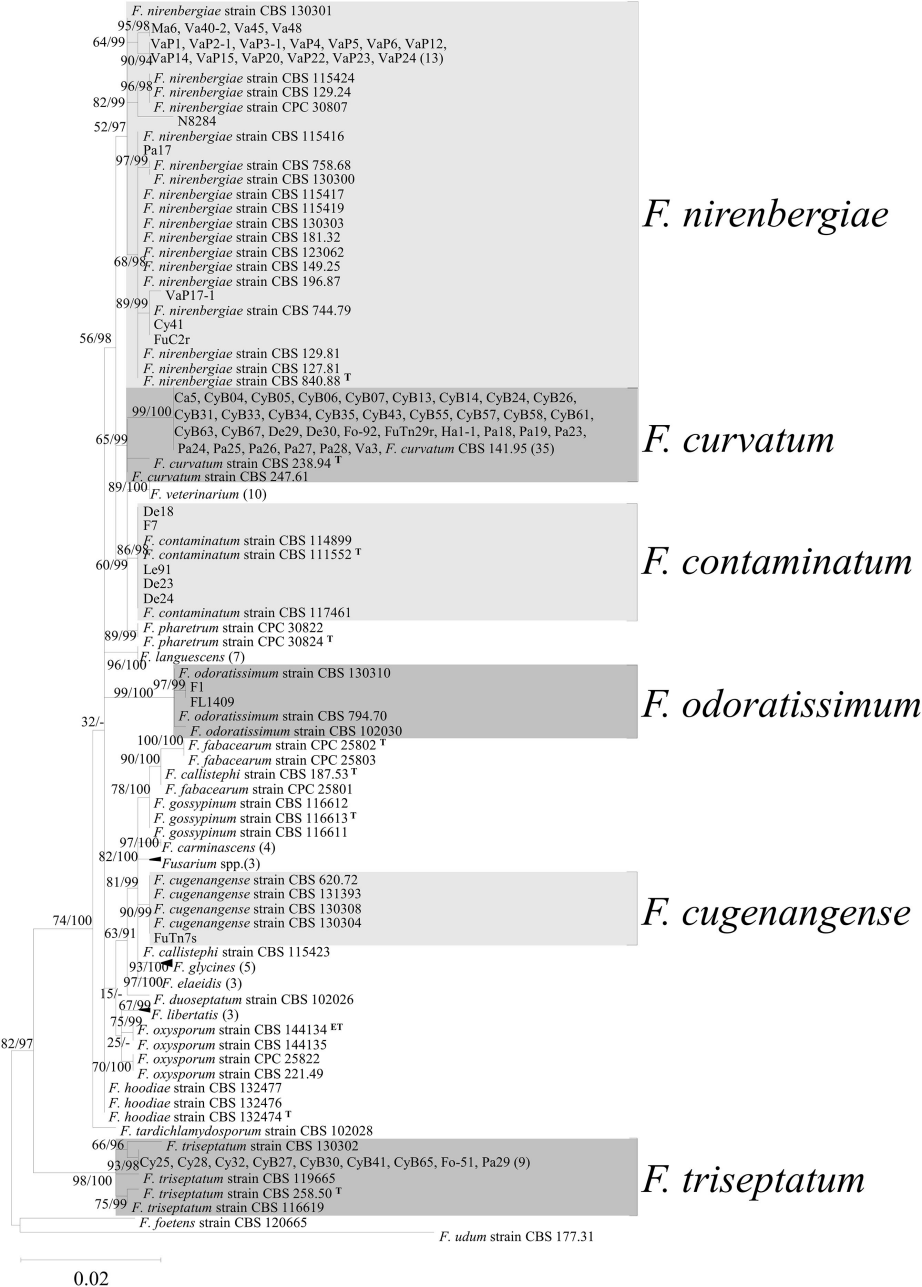
The Maximum Likelihood (ML) phylogenetic tree of *Fusarium oxysporum* species complex (FOSC) isolates from orchid constructed with *tef1* sequence alignment. The K80+G model was applied to the analysis, and ML bootstrap and posterior probability values were indicated on the branches. The scale bar indicates 0.02 changes per site. Triangular symbols indicate collapsed clades that contain multiple isolates, and the numbers in parentheses represent the number of isolates included in each collapsed clade. The 73 isolates from orchids are grouped into six clades. The tree is rooted with *F. foetens* (CBS 120665) and *F. udum* (CBS 177.31). Collapsed clades also include the Ex-type culture for each species. ^ET^: Epitype; ^T^: Ex-type culture.

**Figure 4:**
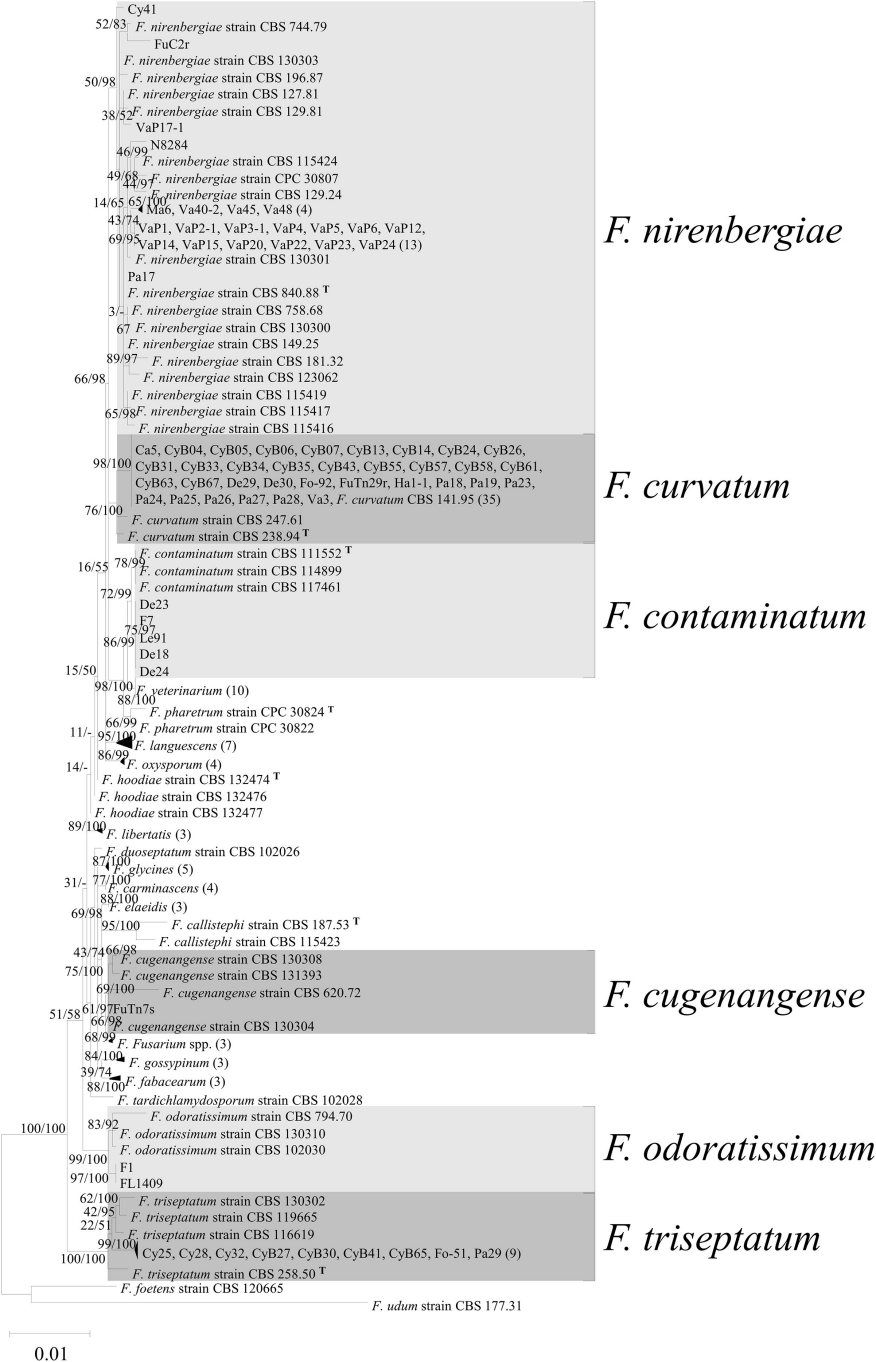
Maximum Likelihood (ML) phylogenetic tree of *Fusarium oxysporum* species complex (FOSC) isolates from different orchids constructed with the combined *cmdA*, *rpb2*, *tef1*, and *tub2* sequence alignment. The K80+G model was selected for the analysis of *tef1* and *tub2*, the K80 model for *cmdA*, and the GTR+G model for *rpb2*. ML bootstrap and posterior probability values are displayed on the branches. The scale bar indicates 0.01 changes per site. Triangular symbols indicate collapsed clades that contain multiple isolates, and the numbers in parentheses represent the number of isolates included in each collapsed clade. The 73 isolates from orchids are grouped into six clades. The tree is rooted with *F. foetens* (CBS 120665) and *F. udum* (CBS 177.31). Collapsed clades also include the Ex-type culture or epitype for each species. ^T^: Ex-type culture.

The test included 63 FOSC isolates obtained from orchids in this study, four isolates from *Anoectochilus formosanus* (*Af*) (F7, Le91, F1, and FL1409) (Huang et al., 2014; Hsu, 2016), two FOSC isolates from *Cy. ensifolium* (Fo-92 and Fo-51) (Huang et al., 2020), and three isolates from *Phalaenopsis* spp. (*Ph*) (FuC2r, FuTn7s, and FuTn29r) supplied by Dr. Wang (Developmental Biology of Phytopathogenic Fungi Lab, National Chung Hsing University, Taiwan), and one isolate from *Ph* (N8284) provided by Dr. Su (Plant Pathology Division, Taiwan Agricultural Research Institute, Taiwan) (Su et al., 2012) were analyzed together (**Supplementary Table S3**). Following the system of Lombard et al. (2019) (**Supplementary Table S2**), the phylogenetic results of the *tef1* single gene can provide an initial classification of FOSC, similar to the results obtained from the multilocus phylogenetic analysis. The 73 FOSC isolates were divided into six taxa (**Figure 3**), including *F. contaminatum* (five isolates), *F. cugenangense* (one isolate), *F. curvatum* (34 isolates), *F. nirenbergiae* (22 isolates)*, F. odoratissimum* (two isolates), and *F. triseptatum* (9 isolates), based on the *tef1* gene sequence (**Table 3**). Isolates grouped with *F. contaminatum* were obtained from *Af* (two isolates) and *De* (three isolates). *Fusarium curvatum* isolates were the dominant species and were obtained from most orchid species, including *Cat, Cy, De, Ha, Pa, Ph*, and *Va*, with the majority being accounted for by *Cy* (20 isolates). Isolates grouped with *F. nirenbergiae* were obtained from *Cy* (one isolate)*, Maxillaria* (*Ma*) (one isolate), *Pa* (one isolate)*, Ph* (two isolates), *Va* (three isolates), and *Vap* (14 isolates), which had the second-highest orchid species diversity. Isolates classified as *F. odoratissimum* were isolated only from *Af* (two isolates). The isolates classified as *F. triseptatum* were from *Cy* (eight isolates) and *Pa* (one isolate). To achieve a more precise classification of these isolates, isolates belonging to different putative *Fusarium* species were used to amplify three additional gene sequences (*cmdA*, *tub2*, and *rpb2*), and phylogenetic analysis was performed based on the four gene sequences. The multigene alignment length was 2,234 bases (*cmdA*, 573 bases; *tef1*, 629 bases; *tub2*, 430 bases; and *rpb2*, 602 bases). The Maximum Likelihood tree is shown in **Figure 4**, and the calculated bootstrap and posterior probability values are shown in the branches. The results indicated that the 73 FOSC isolates used in this study were separated into six taxa, similar to the *tef1* gene sequence analysis (**Table 3**). The GenBank accession number of FOSC isolates from orchid were listed in **Supplementary Table S3**.

**Table 3:** The orchid hosts and number of FOSC isolates in this study based on phylogenetic analyses.

Species^a^	Host	Number of isolation^b^
*F. contaminatum*	*Anoectochilus formosanus*, *Cymbidium*, *Dendrobium*	5
*F. cugenangense*	*Phalaenopsis*	1
*F. curvatum*	*Cattleya*, *Cymbidium*, *Dendrobium*, *Haraella retrocalla*, *Pahiopedilum*, *Phalaenopsis*, *Vanda*	34
*F. nirenbergiae*	*Cymbidium*, *Maxillaria*, *Paphiopedilum*, *Phalaenopsis*, *Vanda*, *Vanilla planifolia*	22
*F. odoratissimum*	*A. formosanus*	2
*F. triseptatum*	*Cymbidium, Paphiopedilum*	9

a Species identification was preliminary based on the phylogenetic result of *tef1*, while subsequent multilocus phylogenetic result of *cmdA*, *tef1*, *tub2*, and *rpb2* showed the same classification.

b The isolates were all subjected to Koch’s postulates, and their pathogenicity was successfully confirmed.

### 3.4 Morphological characteristics of isolates

**Figure 5:**
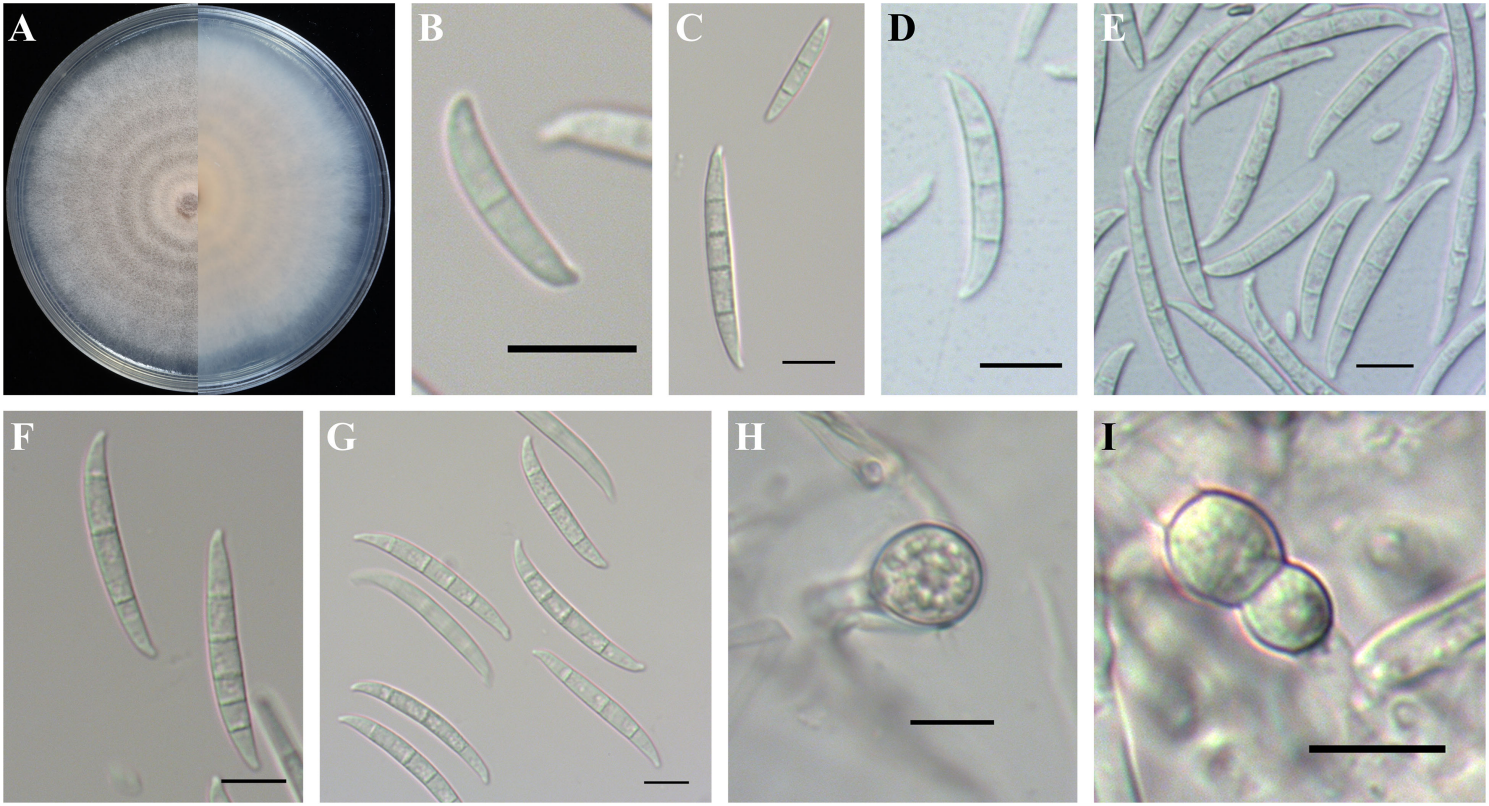
Colony and spore morphology of *Fusarium contaminatum*. **(A)** Colony on potato dextrose agar (PDA); **(B, E)** Microconidia; **(C–G)** Macroconidia; **(H, I)** Chlamydospores. Scale bars = 10 μm.

Based on phylogenetic inference, the existing FOSC (six species) were determined by multilocus phylogenetic analysis and their morphological characteristics are described below. In this study, *F. contaminatum* formed white to bright orange colonies on PDA, and aerial mycelia were abundant (**Figure 5A**). Microconidia were ellipsoidal or falcate-shaped with 0–1 septa, forming false heads, 5.0-(6.6)-14.8 µm in length and 1.8-(2.5)-3.7 µm in width (**Figures 5B–E**). Macroconidia were falcate with slightly curved and foot-like cells with 2–5 septa, 26.0-(35.5)-47.1 µm in length and 2.8-(3.8)-4.8 µm in width (**Figures 5C–G**). Chlamydospores grew singly or in pairs, formed in hyphae and in conidia, mostly globose or subglobose, smooth to rough-walled, 6.0-(9.6)-15.1 µm (**Figures 5H, I**).

**Figure 6:**
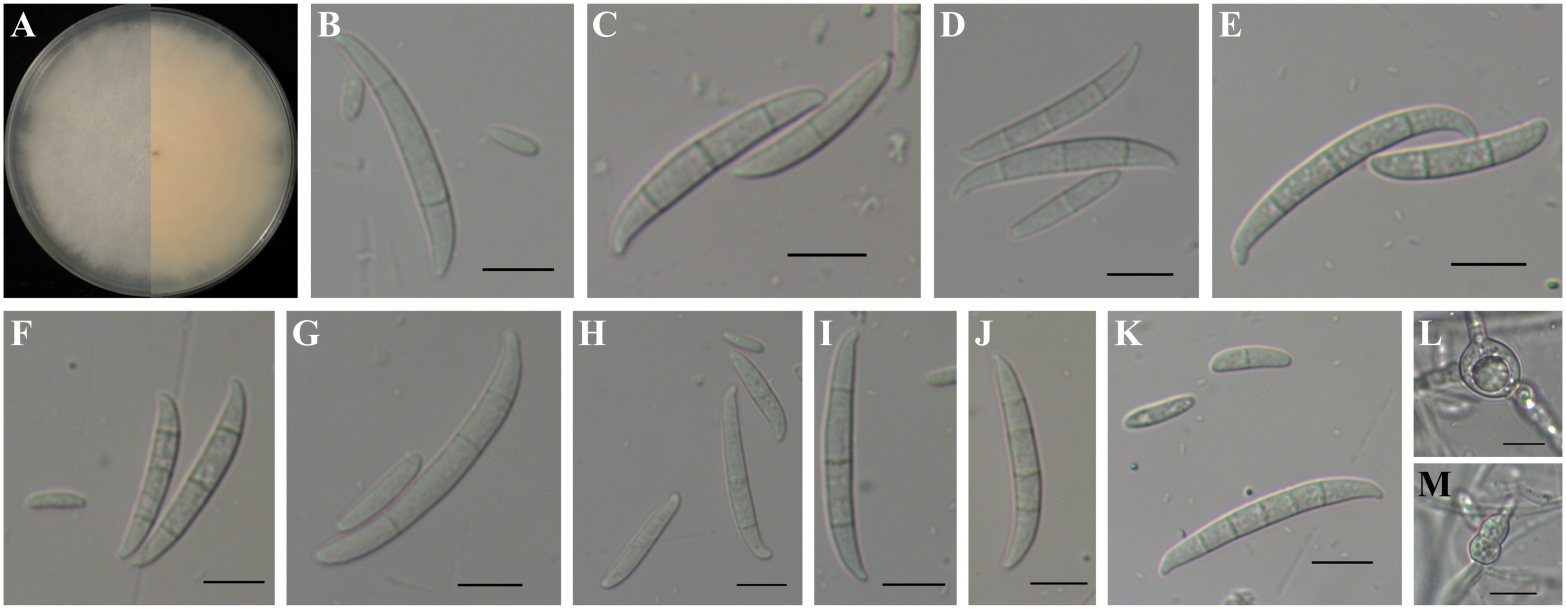
Colony and spore morphology of *Fusarium cugenangense*. **(A)** Colony on potato dextrose agar (PDA); **(B, D, F-H, K)** Microconidia; **(B–K)** Macroconidia; **(L, M)** Chlamydospores. Scale bars = 10 μm.

*Fusarium cugenangense* formed pink to bright purple colonies with less abundant aerial mycelia on PDA, and its color on the reverse side was pink to orange (**Figure 6A**). Microconidia were ellipsoidal or falcate-shaped with 0–1 septa, forming false heads, 7.4-(14.4)-37.5 µm in length and 2.1-(3.0)-4.4 µm in width (**Figures 6B, D, F–H, K**). It had the largest microconidia on CLA among the species mentioned in this study. Microconidia with 1-septa were more abundant than the other species mentioned in this study (**Figures 6B–K**). Macroconidia were falcate with more pronouncedly curved and foot-like cells with 1–5 septa, 22.8-(31.7)-39.0 µm in length and 3.4-(4.0)-4.8 µm in width (**Figures 6C–G**). The 4-septate macroconidia were barely observable (**Figures 6J**). Chlamydospores grew singly or in pairs, formed in hyphae and in conidia, mostly globose or subglobose, smooth to rough-walled, 6.6-(10.7)-21.1 µm (**Figures 6L, M**). It had the largest chlamydospores among the species examined in this study.

**Figure 7:**
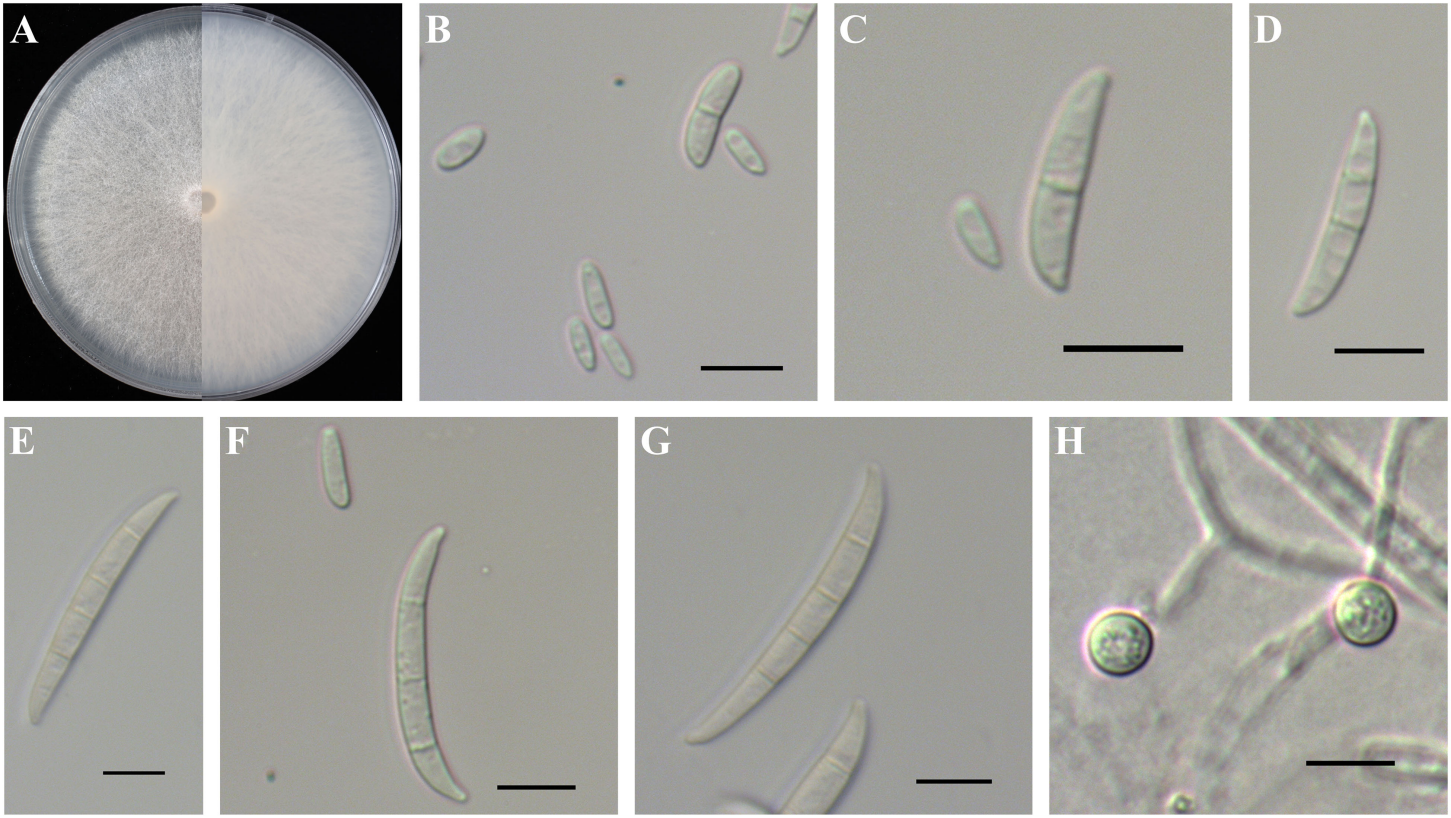
Colony and spore morphology of *Fusarium curvatum*. **(A)** Colony on potato dextrose agar (PDA); **(B, C, F)** Microconidia; **(D–G)** Macroconidia; **(H)** Chlamydospores. Scale bars = 10 μm.

*Fusarium curvatum* was a white to bright pink colony with fewer aerial mycelia than the other species mentioned in this study (**Figure 7A**). Microconidia were ellipsoidal or falcate-shaped with 0–1 septa, forming false heads, 5.5-(7.5)-11.6 µm in length and 1.8-(2.5)-3.4 µm in width (**Figures 7B–C, F**). Macroconidia were falcate with slightly curved and foot-like cells with 2–4 septa, 23.7-(30.1)-36.0 µm in length and 2.7-(3.6)-4.4 µm in width (**Figures 7D–G**). Macroconidia were more curved than those of *F. contaminatum* but not as curved as described by Lombard et al. (2019). Macroconidia of *Fusarium curvatum* were easily produced on CLA; however, 4-septate macroconidia were barely observed. Chlamydospores grew singly, formed in hyphae and in conidia, were mostly globose or subglobose, smooth to rough-walled, 6.0-(6.8)-7.8 µm (**Figures 7H**).

**Figure 8:**
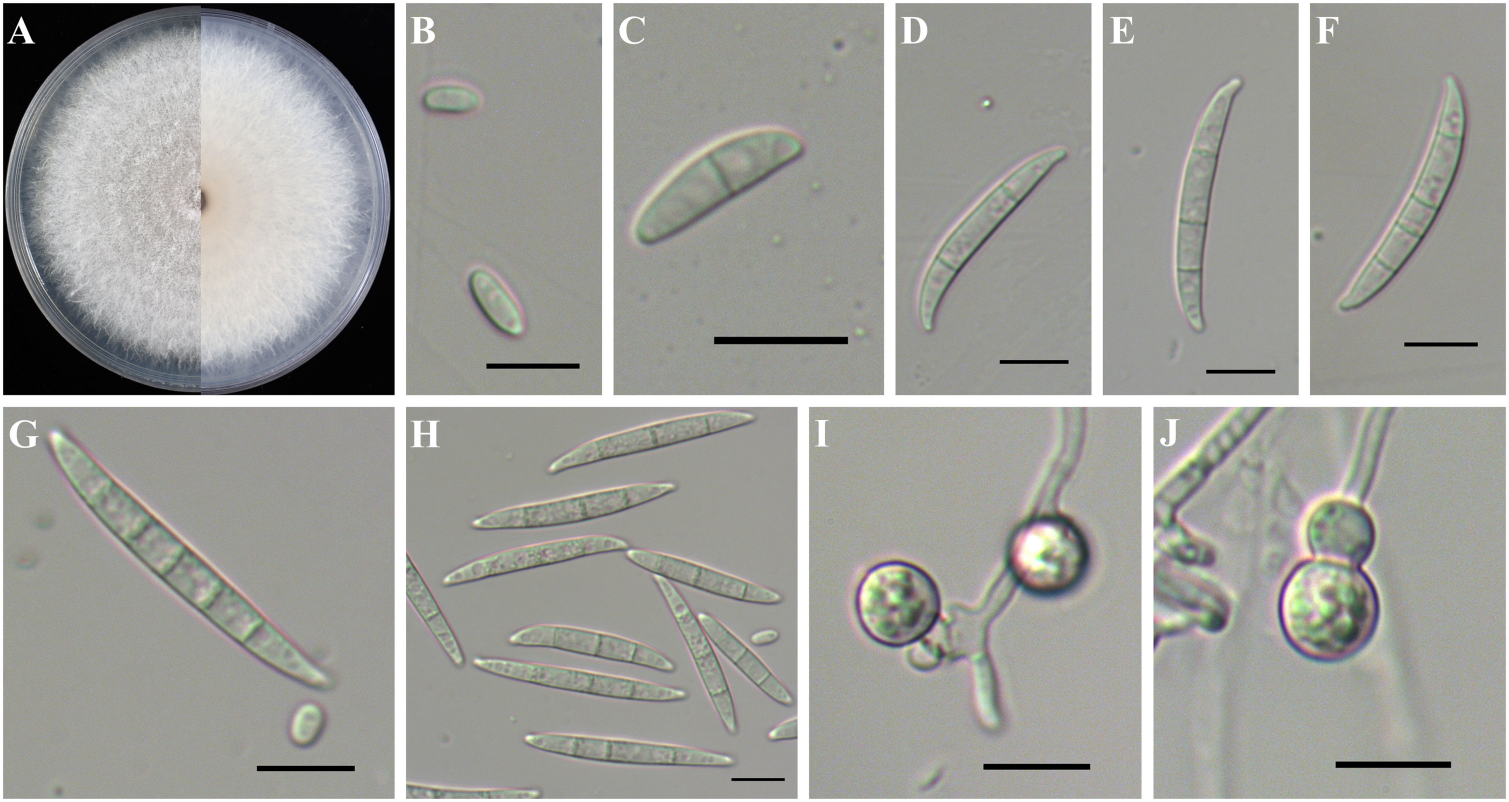
Colony and spore morphology of *Fusarium nirenbergiae*. **(A)** Colony on potato dextrose agar (PDA); **(B, C, G)** Microconidia; **(D–H)** Macroconidia; **(I, J)** Chlamydospores. Scale bars = 10 μm.

*Fusarium nirenbergiae* sometimes formed white to bright purple or bright orange colonies (**Figures 8A**). Although it had fewer aerial mycelia, it also had more aerial mycelia than *F. curvatum* (**Figure 8A**). Microconidia were ellipsoidal, kidney-, or falcate-shaped with 0–1 septa, forming false heads, 4.8-(7.2)-10.6 µm in length and 1.9-(2.5)-3.3 µm in width (**Figures 8B, C, G**). Macroconidia were falcate with slightly curved and foot-like cells with 2–5 septa, 28.2-(36.2)-43.7 µm in length and 2.9-(3.9)-4.7 µm in width (**Figures 8D–H**). Chlamydospores grew singly or in pairs, formed in hyphae and in conidia, mostly globose or subglobose, smooth to rough-walled, 5.5-(7.7)-8.7 µm (**Figures 8I, J**).

**Figure 9:**
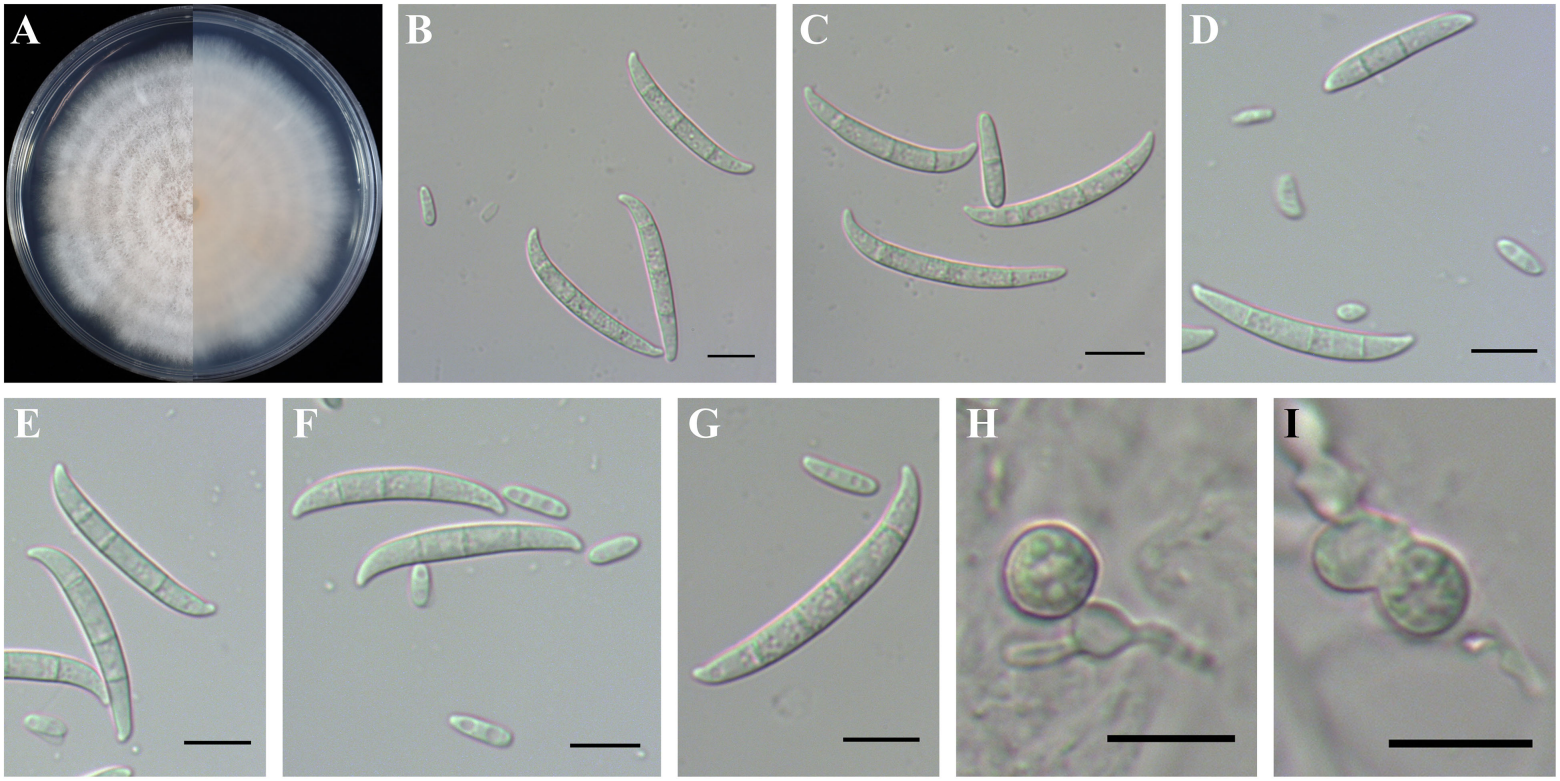
Colony and spore morphology of *Fusarium odoratissimum*. **(A)** Colony on potato dextrose agar (PDA); **(B–G)** Microconidia and macroconidia; **(H, I)** Chlamydospores. Scale bars = 10 μm.

*Fusarium odoratissimum* had white to bright orange colonies, but the aerial mycelia were less abundant than those of *F. contaminatum* (**Figure 9A**). Microconidia were ellipsoidal or kidney-shaped with 0–1 septa, forming false heads, 5.2-(7.9)-10.8 µm in length and 2.1-(2.8)-3.8 µm in width (**Figures 9B–G**). Macroconidia were falcate, slightly curved, wider in width, and had foot-like cells, with 2–4 septa, 27.5-(34.2)-43.2 µm in length and 3.6-(4.2)-5.1 µm in width (**Figures 9B–G**). In the present study, the conidia of *F. odoratissimum* isolates did not possess many septa, as reported by Maryani et al. (2019). Chlamydospores grew singly or in pairs, formed in hyphae and in conidia, mostly globose or subglobose, smooth to rough-walled, 5.8-(7.1)-8.9 µm (**Figures 9H, I**).

**Figure 10:**
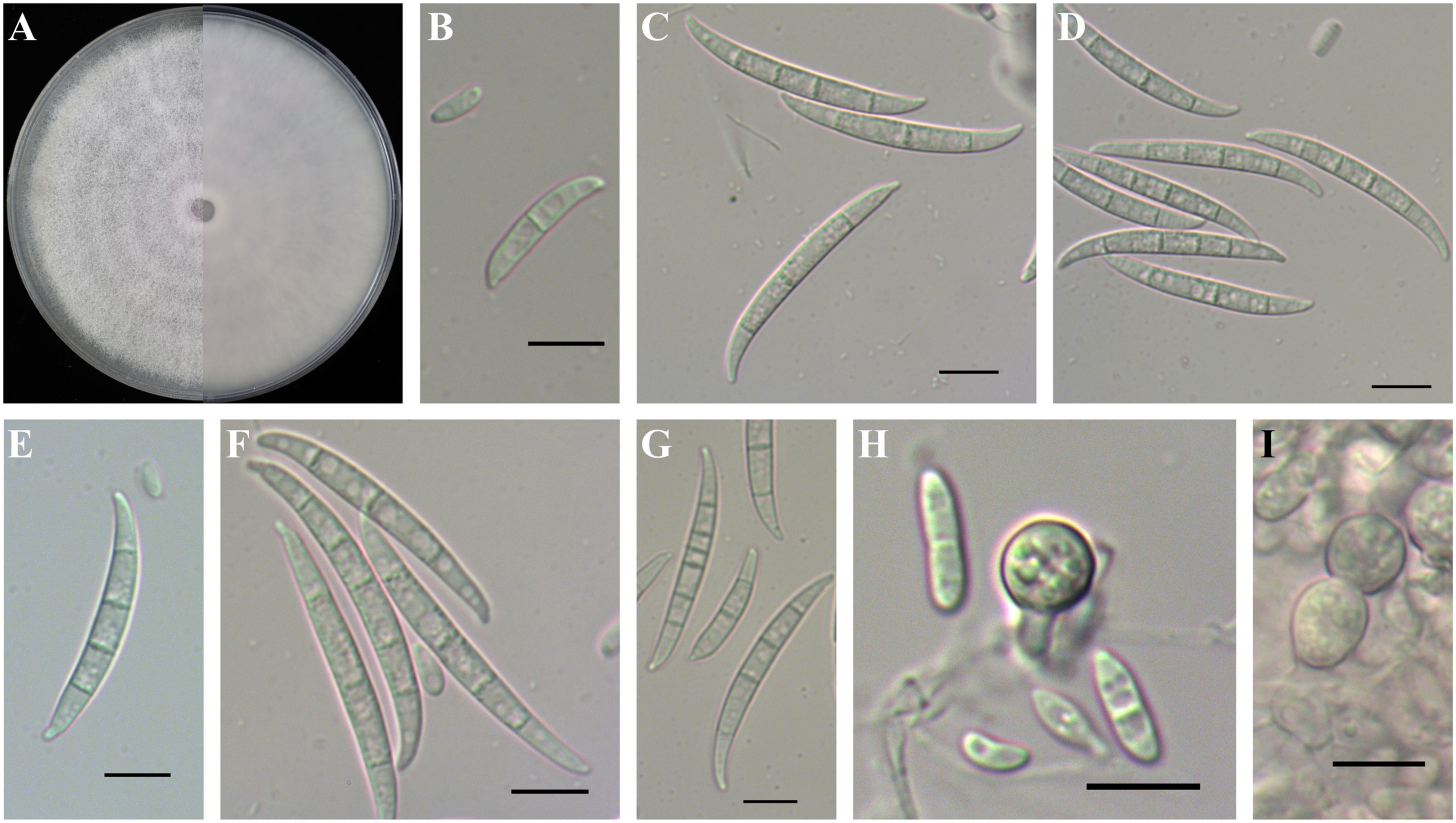
Colony and spore morphology of *Fusarium triseptatum*. **(A)** Colony on potato dextrose agar (PDA); **(B, H)** Microconidia; **(C–G)** Macroconidia; **(H, I)** Chlamydospores. Scale bars = 10 μm.

*Fusarium triseptatum* was purple, with the most abundant aerial mycelia on PDA (**Figure 10A**). Microconidia were ellipsoidal or falcate-shaped with 0–1 septa, forming false heads, 5.2-(7.5)-10.5 µm in length and 1.8-(2.9)-4.3 µm in width (**Figures 10B, F**). Macroconidia were falcate with slightly curved and foot-like cells with 2–6 septa, 20.2-(34.4)-52.6 µm in length and 3.1-(4.3)-5.1 µm in width (**Figures 10C–G**). Among the six FOSC taxa in this study, *F. triseptatum* had the largest and most abundant septa macroconidia on CLA. Chlamydospores grew singly or in pairs, formed in hyphae and in conidia, mostly globose or subglobose, smooth to rough-walled, 6.5-(8.2)-9.7 µm (**Figures 10H, I**).

## 4 Discussion

Symptomatic orchids were collected from horticultural facilities and flower markets. Most cultivars are common in Taiwan. Although our investigation did not encompass all cultivars, it is still fairly representative and provides new insights into orchid diseases. The results indicated that *Cat*, *Cy, De*, *Ha*, *Ma*, *Pa*, *Va*, and *Vap* were the hosts of FOSC. Based on information regarding orchid diseases caused by *Fusarium* spp., Srivastava et al. (2018) concluded that FOSC can cause diseases in *Af*, *Cat*, *Cy*, *De*, *Miltonia*, *Ph*, and *Vap*. A comparison of our results with those of Srivastava et al. (2018) indicates that FOSC have many hosts in Taiwan. Taiwanese FOSC isolates can also cause diseases in *Ha*, *Ma*, *Pa*, and *Va*. In Taiwan, over 477 orchid species have been recorded (Lin, 2024), and highly diverse orchids and cultivar development have a greater chance of allowing FOSC isolates to cause disease in different orchid plants. These findings offer a preliminary understanding of pathogenic FOSC in orchids and may serve as a basis for future research on developing resistant cultivars or identifying effective disease management strategies, such as biocontrol microorganism screening.

Among these, FOSC isolates from *Vap* have been studied for classification and investigation. In India, FOSC can cause root, stem, and bean rot in *Vap* (Vijayan et al., 2012), and the occurrence of stem rot in *Vap* in Indonesia (Ploetz, 2006; Pinaria et al., 2010) indicates that FOSC might have host specificity to *Vap*. A previous study has indicated that FOSC isolates from *Vap* were identified as *F. oxysporum* f. sp. *radicis*-*vanillae* (Koyyappurath et al., 2016). Our results revealed a high uniformity of isolates from *Vap* compared with those from other orchids. These results support the hypothesis that Taiwanese FOSC isolates from *Vap* have host specificity. In the future, the host range of the FOSC isolates from *Vap* should be confirmed.

From molecular phylogenetic analyses, the FOSC isolates obtained from orchids were separated into six species based on *cmdA*, *rpb2*, *tef1*, and *tub2* sequences. *Fusarium oxysporum* is a species complex that can cause wilting, root rot, stem rot or fruit rot in over 120 plant species (van Dam et al., 2016). Among these FOSC, certain isolates have host specificity and have been identified as *formae* sp*eciales* (Gordon, 1965); more than 106 *formae* sp*eciales* have been recorded (Edel-Hermann and Lecomte, 2019). However, Lombard et al. (2019) renamed these FOSC based on phylogenetic analysis. Consequently, the FOSC isolates from the orchid genera were identified as *F. contaminatum*, *F. cugenangense*, *F. curvatum*, *F. nirenbergiae*, *F. odoratissimum*, and *F. triseptatum*.

*Fusarium contaminatum* is a contaminant of fruit juice, chocolate milk, or pack milky (Lombard et al., 2019). No information is available on whether this species is a pathogen that infects crops. *Fusarium contaminatum* could be obtained from *Af* and *De* and causes stem blight, stem rot, or pseudobulb rot. Thus, this species is not only a contaminant in food but also a pathogen in crops such as orchids. Although this species is not dominant, we need to pay attention to it.

In a previous study published by Lombard et al. in 2019, *F. cugenangense* contained two formae speciales, *F. oxysporum* f. sp. *gladioli* and f. sp. *vasinfectum*, and the hosts included *Crocus* sp., *Gossypium barbadense*, and *Vicia faba.* One isolate of this species was collected from a human toenail. Recently, more records of this species as a pathogen have been published, including *Fusarium* wilt on Korean blackberries and *Pyrus pyrifolia* (Kim et al., 2021; Li et al., 2024). It also causes root rot in tea (*Camellia sinensis*) and strawberries (Shrestha et al., 2024; Yang Y. et al., 2024). In the present study, only FuTn7s isolated from *Phalaenopsis* sp. was classified as *F. cugenangense*. *Phalaenopsis* is the primary plant in Taiwan. However, FOSC are minor pathogens in *Phalaenopsis* compared to *F. phalaenopsidis* belonging to *Fusarium solani* species complex (Chung et al., 2011; Tsao et al., 2024). Because pathogenic isolates were unavailable in this investigation, four isolates were borrowed and analyzed. Although only one isolate was classified as this species in this study, its potentially wide host range deserves attention.

*Fusarium curvatum* includes two formae speciales, *F. oxysporum* f. sp. *matthiolae* and f. sp. *meniscoideum*, and its hosts include *Mattiola incana*, *Beaucamia* sp., and *Hedera helix* (Lombard et al., 2019). Moreover, *F. curvatum* has been reported to cause leaf spots in cherry (Zhou et al., 2022) and wilt in lettuce (Claerbout et al., 2023). Importantly, this species has been shown to cause dieback disease, resulting in the death of the tips on *Dendrobium* in China (Mirghasempour et al., 2022). In contrast, the rot symptoms in *Dendrobium* that we collected most frequently emerged in the middle section of the stem, and these seemed like some of their diseased samples. However, the colonial morphology of *F. curvatum* isolates differed between the two. Additionally, *F. curvatum* showed a smaller proportion of *Dendrobium*, similar to the results obtained in China (Mirghasempour et al., 2022). In this study, this species was obtained not only from *De*, also from *Cat*, *Cy*, *De*, *Ha*, *Pa*, *Ph*, and *Va*. As a result, it was found to be the dominant species among the orchids. In Taiwan, no information indicates that *F. oxysporum* causes diseases in *M. incana*, *Beaucamia* sp., *H. helix*, or cherries. The pathogenicity of *F. curvatum* from orchids could try to inoculate in these hosts to confirm whether *F. curvatum* has pathogenicity in these four hosts in the future. This species also causes human fusariosis in Taiwan (Lu et al., 2023). The potential threats to field managers cannot be ignored.

*Fusarium nirenbergiae* includes seven formae speciales with 10 hosts, including *Musa* sp. (f. sp. *cubense*), *Solanum lycopersicum* (f. sp. *lycopersici* and f. sp. *radices-lycopersici*), *Passiflora edulis* (f. sp. *passiflorae*), *Dianthus caryophyllus* (f. sp. *dianthi*), and *Chrysanthemum* sp. (f. sp. *chrysanthemi*), and is a pathogen in humans (Lombard et al., 2019). In Taiwan, *F. oxysporum* f. sp. *cubense*, f. sp. *lycopersici*, f. sp. *Dianthi*, and f. sp. *chrysanthemi* have been reported, especially, f. sp. *cubnese* and f. sp. *lycopersici* are common in field. Additionally, *F. nirenbergiae* is a pathogen that causes keratitis in humans (Huang et al., 2022). Thus, *F. nirenbergiae* has a wide host range and can infect both plants and humans. In this study, orchid isolates identified as *F. nirenbergiae* were obtained from *Cy*, *Ma*, *Pa*, *Ph*, *Va*, and *Vap*. In contrast, Mirghasempour et al. (2022) reported that *F. nirenbergiae* can cause disease in *Dendrobium* and revealed that *F. nirenbergiae* is a common species that cause disease in *Dendrobium*. However, no *F. nirenbergiae* isolate was collected from *De* in this study. The FOSC isolates from *Vap* were only categorized as *F. nirenbergiae*. Based on the *tef1* sequence, Taiwanese FOSC isolates from *Vap* form a monophylogeny with isolates from Indonesia (Pinaria et al., 2015) (**Supplementary Figure S1**). In Taiwan, *Vap* was introduced from Indonesia in 2006. This might explain why Taiwanese FOSC isolates from *Vap* grouped with Indonesian isolates. However, Flores-de la Rosa et al. (2018) have reported that *Vap* isolates from Mexico are polyphylogenic. Our results also indicate that FOSC from *Vap* formed a different subgroup. Therefore, *F. nirenbergiae* has complex hosts, but exhibits host specificity for certain hosts. In the future, it will be necessary to inoculate the *F. nirenbergiae* from orchids into other hosts to confirm their pathogenicity.

*Fusarium odoratissimum* included only two isolates from *Af* formed by this species. Lombard et al. (2019) have reported that isolates from *Musa* spp. (f. sp. *cubense*) and *Albizzia julibrissin* (f. sp. *perniciosum*) belong to *F. odoratissimum*. A previous study has reported that *F. oxysporum* isolates from *Af* could be separated into two colony types (cottony alba type and sporodochial type), and the cottony alba type showed higher virulence than the sporodochial type (Huang et al., 2014). *Fusarium odoratissimum* isolates (F1 and FL1409) belong to the cottony alba type, which has high virulence in *Af*. *Fusarium oxysporum* f. sp. *cubense* is polyphylogenic, with nine phylogenetic species (Lombard et al., 2019; Maryani et al., 2019), similar to the isolates from *Af* which have different virulence in *Af*.

*Fusarium triseptatum* consists of two formae speciales, *F. oxysporum* f. sp. *batatas* from *Ipomoea batatas* and f. sp. *vasinfectum* from *Gossypium hirsutum*, as well as *F. oxysporum* isolates from sago starch and the human eye (Lombard et al., 2019). This species can cause root rot in cassava (da Silva et al., 2025), and dry rot in carrots (Favaro et al., 2024). In the present study, four isolates from *Cy* and one isolate from *Pa* were formed with *F. triseptatum*. In Taiwan, *F. oxysporum* f. sp. *batatas* has been reported to cause sweet potato wilting (Tzean, 2019); however, it is difficult to detect this pathogen in the field (Chen, 2015). Taken together, these six species may represent potential risk hosts for future investigations. The existing lineages also contribute to a better understanding of FOSC population in orchids and could facilitate the detection and prevention of foreign species.

Cross-infection may occur in FOSC species within the same plant family, such as Cucurbitaceae (van Dam et al., 2016). Huang et al. (2014) also reported that isolates collected from *Af* could cause slight symptoms in *Cy.* This suggests that cross-infection may occur in different orchid plants. Here, we try to figure out the pathogenicity of FOSC isolates from different orchids on *Af*, *Cy*, *De*, *On*, and *Ph* which are important orchids in Taiwan (**Supplementary Figure S2**). The results revealed that some of the isolates from *Cy, De, Pa, Va*, *Vap* could infect *Af* (**Supplementary Figure S2A**); non-*Cy* isolates caused slight symptom on *Cy* (**Supplementary Figure S2B**); some of the isolates from *Af*, *Cy*, *De*, *Pa*, *Va*, and *Vap* could infect *De* (**Supplementary Figure S2C**). Preliminary inoculation tests were conducted on *Ph* and *On*, the orchids with higher commercial value, despite the absence of positive controls (isolates isolated from *Ph* or *On*). In *On* test, some of isolates from *Cy*, *Ha*, *Ma*, *Pa*, *Va*, and *Vap* isolates could cause symptoms, while all isolates from *De* were able to infect *On*. In *Ph* test, non-*Ph* isolates had potential to cause symptoms on *Ph*, and some individual isolates induced severe symptoms (**Supplementary Figure S2E**). Thus, FOSC isolates from orchids can share different hosts. However, due to the variability observed in the inoculation tests, drawing definitive conclusions requires more comprehensive data. Moreover, although host range is usually determined by virulence genes rather than housekeeping genes (van Dam et al., 2016), the phylogenetic results of this study provide a potential relationship between pathogenic FOSC and different orchids. In the future, non-original host inoculations will be conducted more comprehensively to study the characteristics of these pathogens. Additionally, it is necessary to study the virulence-related effectors of FOSC.

## Data Availability

The datasets presented in this study can be found in online repositories. The names of the repository/repositories and accession number(s) can be found in the article/**Supplementary Material**.
