# Supplementary material for: Orchid diseases caused by Fusarium oxysporum species complex in Taiwan
Source: Front Plant Sci. 2026 Jan 19;16:1630094. doi: 10.3389/fpls.2025.1630094 (PMC12863263; doi:10.3389/fpls.2025.1630094)
Supplement: Supplementary file 1 [file DataSheet1.docx]

Supplementary Material

Orchid Diseases Caused by *Fusarium oxysporum* Species Complex (FOSC) in Taiwan

**An Chang, Che-Wei Chang, Cheng-Chun Wu, Kuo-Hsi Lin, Nittaya Chookoh, Jintana Unartngam, Wen-Hsin Chung*

Correspondence:**
Wen-Hsin Chung, Department of Plant Pathology, National Chung Hsing University, Taichung 40227, Taiwan.
Email: wenchung@nchu.edu.tw

# Supplementary Data

# Supplementary Figures and Tables

## Supplementary Figures


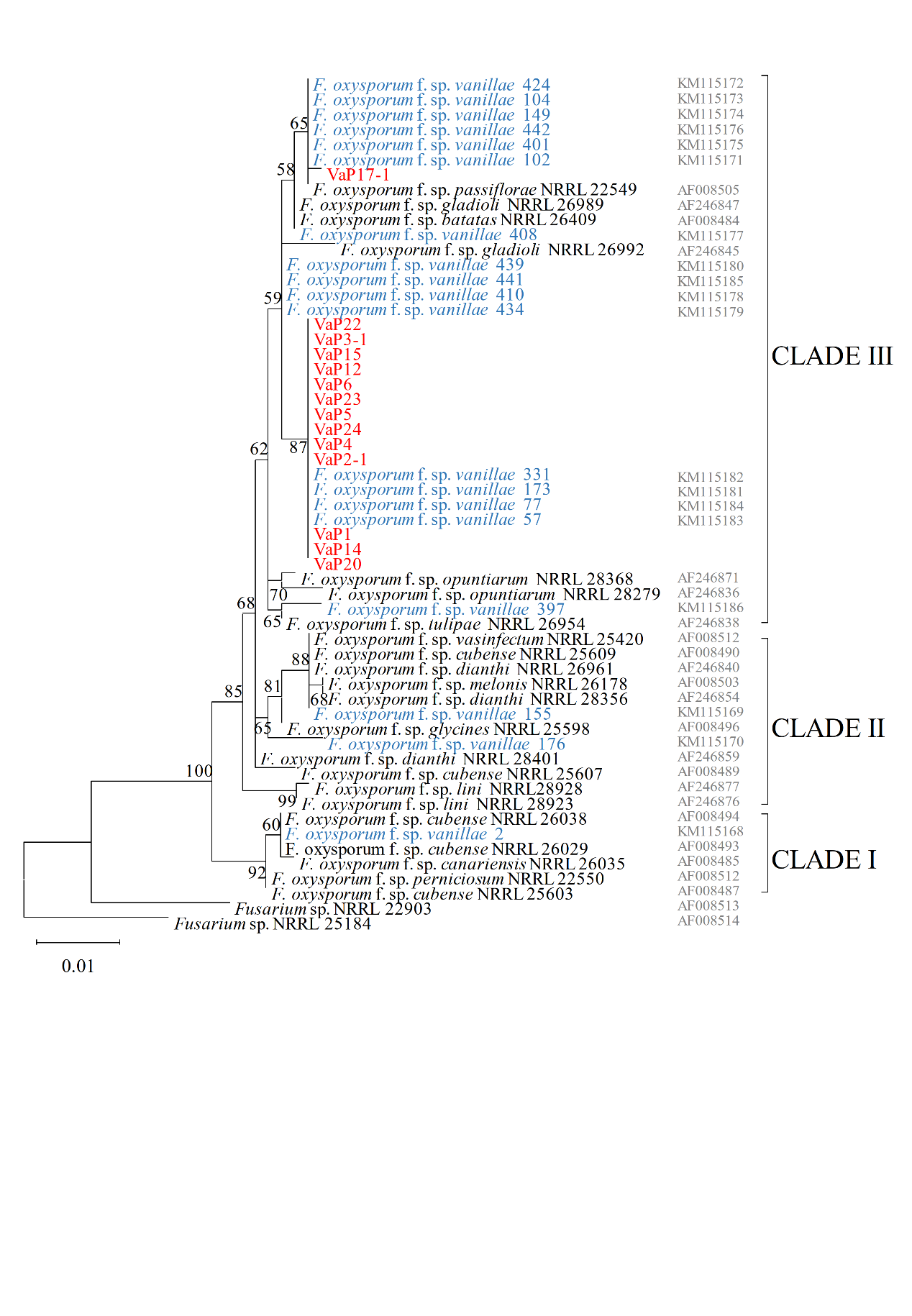


**Supplementary Figure 1.** The Maximum Likelihood (ML) phylogenetic tree of *Fusarium oxysporum* species complex (FOSC) isolates from *Vanilla planifolia* constructed by *tef1* sequence alignment. A K80+G model is selected for analysis, and ML bootstrap is indicated at the branches. The scale bar indicates 0.01 changes per site. The 19 isolates from *V. planifolia* in Pinaria et al., 2015 (marked in blue) could be divided into three clades, and the 14 isolates (marked in red) in this study were grouped in CLADE III. The tree is rooted with *Fusarium* sp. (NRRL 22903 and NRRL 25184).

**
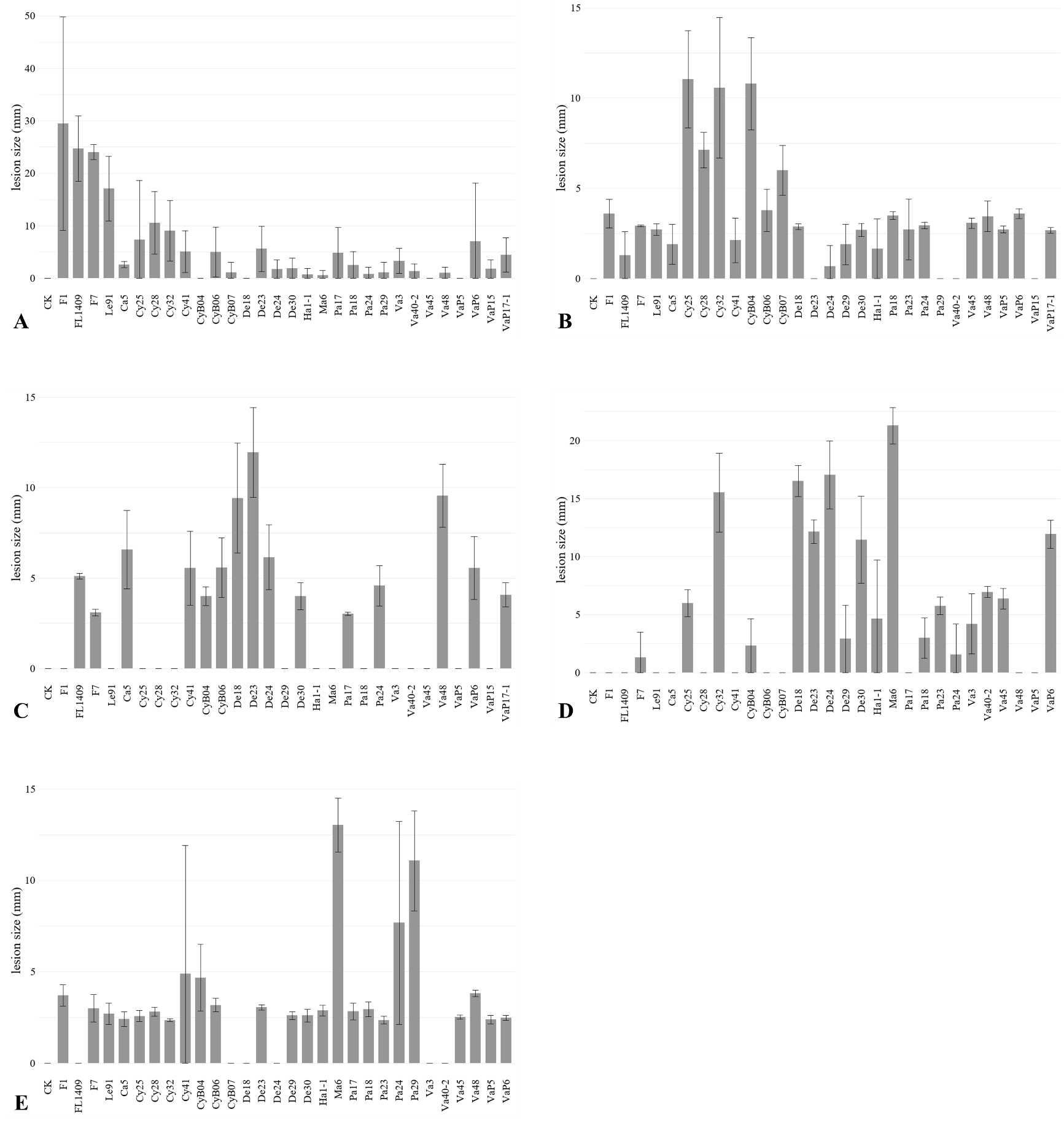
Supplementary Figure 2.** The preliminary cross-inoculation results on five major orchid species. Isolates from different orchids were inoculated on five major orchid species, including *Anoectochilus formosanus* (*Af*), *Cymbidium* (*Cy*), *Dendrobium* (*De*), *Phalaenopsis* (*Ph*), and *Oncidium* (*On*). Spore suspension was adjusted to 1 × 10^7^ spores/mL, and mixed 1:1 (v/v) with 0.2% WA. Sterilized ddH_2_O was used as negative control. Four wounds were inoculated with 10 μL of spore suspension in *Cy* and *Ph*, and with 20 μL in *Af*, *De*, and *On*. Lesion sizes were recorded at 7d after inoculation (DAI) in *Ph*, at 10 DAI in *Af* and *Cy*, and at 14 DAI in *De* and *On.* (**A**) Inoculation result on *Af*; (**B**) on *Cy*; (**C**) on *De*; (**D**) on *On*; (**E**) on *Ph***.**

## Supplementary Tables

**Supplementary Table 1. The list of primers used in this study**

| **Target** | **Primer name** | **Sequences** | **Reference** | **Program** |
| --- | --- | --- | --- | --- |
| *cmdA* | Cal228F | 5’-GAGTTCAAGGAGGCCTTCTC-3’ | Carbone and Kohn, 1999 | 96℃ 5 min; 96℃ 30 sec, 52℃ 30 sec, 72℃ 1 min (30 cycles); 72 ℃ 5 min |
|  | CAL2Rd | 5’-TGRTCNGCCTCDCGGATCATCTC-3’ | Carbone and Kohn, 1999 |  |
| *rpb2* | RPB2-F | 5’-CGAACCAATACTCCCATC-3’ | Wu, 2023 | 95℃ 5 min; 95℃ 1 min, 54℃ 2 min, 72℃ 2 min (30 cycles); 72℃ 10 min |
|  | RPB2-R | 5’-TGGCACAGATACCGAGAA-3’ | Wu, 2023 |  |
| *tef1* | EF1 | 5’-ATGGGTAAGGARGACAAGAC-3’ | O’Donnell et al., 1998 | 94℃ 2 min; 94℃ 1 min, 60℃ 90 sec, 72℃ 2 min (35 cycles); 72 ℃ 10 min |
|  | EF2 | 5’-GGARGTACCAGTSATCATGTT-3’ | O’Donnell et al., 1998 |  |
| *tub2* | T1 | 5'-AACATGCGTGAGATTGTAAGT -3' | O’Donnell and Cigelnik, 1997 | 95℃ 8 min; 95℃ 30 sec, 55℃ 45 sec, 72℃ 1 min (35 cycles); 72℃ 5 min |
|  | CYLTUB1R | 5'-AGTTGTCGGGACGGAAGAG-3' | Crous et al., 2004 |  |

**Supplementary Table 2.** The list of *Fusarium* *oxysporum* species complex isolates in Lombard et al., 2019 were used to conduct multilocus phylogenetic analysis.

| **Species** | **Isolate code** | **Source** | **Origin** | **GenBank accession number**^a^ | | | |
| --- | --- | --- | --- | --- | --- | --- | --- |
|  |  |  |  | ***cmdA*** | ***tub2*** | ***rpb2*** | ***tef1*** |
| ***F. callistephi*** | CBS 187.53 | *Callistephus chinensis* | The Netherlands | MH484693 | MH485057 | MH484875 | MH484966 |
|  | CBS 115423 | *Agathosma betulina* | South Africa | MH484723 | MH485087 | MH484905 | MH484996 |
| ***F. carminascens*** | CPC 25792 | *Zea mays* | South Africa | MH484752 | MH485116 | MH484934 | MH485025 |
|  | CPC 25793 | *Z. mays* | South Africa | MH484753 | MH485117 | MH484935 | MH485026 |
|  | CPC 25795 | *Z. mays* | South Africa | MH484754 | MH485118 | MH484936 | MH485027 |
|  | CPC 25800 | *Z. mays* | South Africa | MH484755 | MH485119 | MH484937 | MH485028 |
| ***F. contaminatum*** | CBS 111552 | Pasteurized fruit juice | The Netherlands | MH484718 | MH485082 | MH484900 | MH484991 |
|  | CBS 114899 | Pasteurized chocolate milk | Germany | MH484719 | MH485083 | MH484901 | MH484992 |
|  | CBS 117461 | Tetra pack with milky nutrition | The Netherlands | MH484729 | MH485093 | MH484911 | MH485002 |
| ***F. cugenangense*** | CBS 620.72 | *Crocus* sp. | Germany | MH484697 | MH485061 | MH484879 | MH484970 |
|  | CBS 130304 | *Gossypium barbadense* | China | MH484739 | MH485103 | MH484921 | MH485012 |
|  | CBS 130308 | Human toe nail | New Zealand | MH484738 | MH485102 | MH484920 | MH485011 |
|  | CBS 131393 | *Vicia faba* | Australia | MH484746 | MH485110 | MH484928 | MH485019 |
| ***F. curvatum*** | CBS 247.6 | *Matthiola incana* | Germany | MH484694 | MH485058 | MH484876 | MH484967 |
|  | CBS 238.94 | *Beaucarnia* sp. | The Netherlands | MH484711 | MH485075 | MH484893 | MH484984 |
|  | CBS 141.95 | *Hedera helix* | The Netherlands | MH484712 | MH485076 | MH484894 | MH484985 |
| ***F. duoseptatum*** | CBS 102026 | *Musa sapientum* cv. Pisang ambon | Malaysia | MH484714 | MH485078 | MH484896 | MH484987 |
| ***F. elaeidis*** | CBS 217.49 | *Elaeis* sp. | Zaire | MH484688 | MH485052 | MH484870 | MH484961 |
|  | CBS 218.49 | *Elaeis* sp. | Zaire | MH484689 | MH485053 | MH484871 | MH484962 |
|  | CBS 255.52 | *Elaeis guineensis* | Unknown | MH484692 | MH485056 | MH484874 | MH484965 |
| ***F. fabacearum*** | CPC 25801 | *Z. mays* | South Africa | MH484756 | MH485120 | MH484938 | MH485029 |
|  | CPC 25802 | *Glycine max* | South Africa | MH484757 | MH485121 | MH484939 | MH485030 |
|  | CPC 25803 | *Gl. max* | South Africa | MH484758 | MH485122 | MH484940 | MH485031 |
| ***F. glycines*** | CBS 176.33 | *Linum usitatissium* | Unknown | MH484686 | MH485050 | MH484868 | MH484959 |
|  | CBS 214.49 | Unknown | Argentina | MH484687 | MH485051 | MH484869 | MH484960 |
|  | CBS 200.89 | *Ocimum basilicum* | Italy | MH484706 | MH485070 | MH484888 | MH484979 |
|  | CPC 25804 | *Gl. max* | South Africa | MH484759 | MH485123 | MH484941 | MH485032 |
|  | CPC 25808 | *Gl. max* | South Africa | MH484760 | MH485124 | MH484942 | MH485033 |
| ***F. gossypinum*** | CBS 116611 | *Gossypium hirsutum* | Ivory Coast | MH484725 | MH485089 | MH484907 | MH484998 |
|  | CBS 116612 | *Go. hirsutum* | Ivory Coast | MH484726 | MH485090 | MH484908 | MH484999 |
|  | CBS 116613 | *Go. hirsutum* | Ivory Coast | MH484727 | MH485091 | MH484909 | MH485000 |
| ***F. hoodiae*** | CBS 132474 | *Hoodia gordonii* | South Africa | MH484747 | MH485111 | MH484929 | MH485020 |
|  | CBS 132476 | *Ho. gordonii* | South Africa | MH484748 | MH485112 | MH484930 | MH485021 |
|  | CBS 132477 | *Ho. gordonii* | South Africa | MH484749 | MH485113 | MH484931 | MH485022 |
| ***F. languescens*** | CBS 645.78 | *Solanum lycopersicum* | Morocco | MH484698 | MH485062 | MH484880 | MH484971 |
|  | CBS 646.78 | *S. lycopersicum* | Morocco | MH484699 | MH485063 | MH484881 | MH484972 |
|  | CBS 413.90 | *S. lycopersicum* | Israel | MH484708 | MH485072 | MH484890 | MH484981 |
|  | CBS 300.91 | *S. lycopersicum* | The Netherlands | MH484709 | MH485073 | MH484891 | MH484982 |
|  | CBS 302.91 | *S. lycopersicum* | The Netherlands | MH484710 | MH485074 | MH484892 | MH484983 |
|  | CBS 872.95 | *S. lycopersicum* | Unknown | MH484713 | MH485077 | MH484895 | MH484986 |
|  | CBS 119796 | Z. mays | South Africa | MH484735 | MH485099 | MH484917 | MH485008 |
| ***F. libertatis*** | CPC 25782 | *Aspalathus* sp. | South Africa | MH484750 | MH485114 | MH484932 | MH485023 |
|  | CPC 25788 | *Aspalathus* sp. | South Africa | MH484751 | MH485115 | MH484933 | MH485024 |
|  | CPC 28465 | Rock surface | South Africa | MH484762 | MH485126 | MH484944 | MH485035 |
| ***F. nirenbergiae*** | CBS 129.24 | *Secale cereale* | Unknown | MH484682 | MH485046 | MH484864 | MH484955 |
|  | CBS 149.25 | *Musa* sp. | Unknown | MH484683 | MH485047 | MH484865 | MH484956 |
|  | CBS 181.32 | *S. tuberosum* | USA | MH484776 | MH485049 | MH484867 | MH484958 |
|  | CBS 758.68 | *S. lycopersicum* | The Netherlands | MH484786 | MH485059 | MH484877 | MH484968 |
|  | CBS 744.79 | *Passiflora edulis* | Brazil | MH484791 | MH485064 | MH484882 | MH484973 |
|  | CBS 127.81 | *Chrysanthemum* sp. | USA | MH484792 | MH485065 | MH484883 | MH484974 |
|  | CBS 129.81 | *Chrysanthemum* sp. | USA | MH484794 | MH485067 | MH484885 | MH484976 |
|  | CBS 196.87 | *Bouvardia longiflora* | Italy | MH484795 | MH485068 | MH484886 | MH484977 |
|  | CBS 840.88 | *Dianthus caryophyllus* | The Netherlands | MH484796 | MH485069 | MH484887 | MH484978 |
|  | CBS 115416 | *Agathosma betulina* | South Africa | MH484811 | MH485084 | MH484902 | MH484993 |
|  | CBS 115417 | *Ag. betulina* | South Africa | MH484812 | MH485085 | MH484903 | MH484994 |
|  | CBS 115419 | *Ag. betulina* | South Africa | MH484813 | MH485086 | MH484904 | MH484995 |
|  | CBS 115424 | *Ag. betulina* | South Africa | MH484815 | MH485088 | MH484906 | MH484997 |
|  | CBS 123062 | Tulip roots | USA | MH484828 | MH485101 | MH484919 | MH485010 |
|  | CBS 130300 | Amputated human toe | USA | MH484834 | MH485107 | MH484925 | MH485016 |
|  | CBS 130301 | Human leg ulcer | USA | MH484835 | MH485108 | MH484926 | MH485017 |
|  | CBS 130303 | *S. lycopersicum* | USA | MH484832 | MH485105 | MH484923 | MH485014 |
|  | CPC 30807 | Unknown | South Africa | MH484859 | MH485132 | MH484950 | MH485041 |
| ***F. odoratissimum*** | CBS 794.70 | *Albizzia julibrissin* | Iran | MH484787 | MH485060 | MH484878 | MH484969 |
|  | CBS 102030 | *Mu* *sapientum* cv. Pisang mas | Malaysia | MH484807 | MH485080 | MH484898 | MH484989 |
|  | CBS 130310 | *Musa* sp. | Australia | MH484831 | MH485104 | MH484922 | MH485013 |
| ***F. oxysporum*** | CBS 221.49 | *Camellia sinensis* | South East Asia | MH484781 | MH485054 | MH484872 | MH484963 |
|  | CBS 144134 | *S. tuberosum* | Germany | MH484862 | MH485135 | MH484953 | MH485044 |
|  | CBS 144135 | *S. tuberosum* | Germany | MH484863 | MH485136 | MH484954 | MH485045 |
|  | CPC 25822 | *Protea* sp. | South Africa | MH484852 | MH485125 | MH484943 | MH485034 |
| ***F. pharetrum*** | CPC 30822 | *Aliodendron dichotomum* | South Africa | MH484860 | MH485133 | MH484951 | MH485042 |
|  | CPC 3082 | *Ali. dichotomum* | South Africa | MH484861 | MH485134 | MH484952 | MH485043 |
| ***F. trachichlamydosporum*** | CBS 102028 | *Mu. sapientum* cv. Pisang awak legor | Malaysia | MH484806 | MH485079 | MH484897 | MH484988 |
| ***F. triseptatum*** | CBS 258.50 | *Ipomoea batatas* | USA | MH484782 | MH485055 | MH484873 | MH484964 |
|  | CBS 116619 | *Go. hirsutum* | Ivory Coast | MH484819 | MH485092 | MH484910 | MH485001 |
|  | CBS 119665 | Sago starch | Papua New Guinea | MH484825 | MH485098 | MH484916 | MH485007 |
|  | CBS 130302 | Human eye | USA | MH484833 | MH485106 | MH484924 | MH485015 |
| ***F. veterinarium*** | CBS 109898 | Shark peritoneum | The Netherlands | MH484808 | MH485081 | MH484899 | MH484990 |
|  | CBS 117787 | Swab sample near filling apparatus | The Netherlands | MH484821 | MH485094 | MH484912 | MH485003 |
|  | CBS 117790 | Swab sample near filling apparatus | The Netherlands | MH484822 | MH485095 | MH484913 | MH485004 |
|  | CBS 117791 | Pasteurized milk-based product | The Netherlands | MH484823 | MH485096 | MH484914 | MH485005 |
|  | CBS 117792 | Pasteurized milk-based product | The Netherlands | MH484824 | MH485097 | MH484915 | MH485006 |
|  | NRRL 54984 | Mouse mucosa | USA | MH484854 | MH485127 | MH484945 | MH485036 |
|  | NRRL 54996 | Little blue penguin foot | USA | MH484855 | MH485128 | MH484946 | MH485037 |
|  | NRRL 62542 | Unknown animal faeces | USA | MH484856 | MH485129 | MH484947 | MH485038 |
|  | NRRL 62545 | Endoscope of veterinary clinic | USA | MH484857 | MH485130 | MH484948 | MH485039 |
|  | NRRL 62547 | Canine stomach | USA | MH484858 | MH485131 | MH484949 | MH485040 |
| ***Fusarium* sp.** | CBS 128.81 | *Chrysanthemum* sp. | USA | MH484793 | MH485066 | MH484884 | MH484975 |
|  | CBS 680.89 | *Cucumis sativus* | The Netherlands | MH484798 | MH485071 | MH484889 | MH484980 |
|  | CBS 130323 | Human nail | Australia | MH484836 | MH485109 | MH484927 | MH485018 |
| ***F. foetens***^b^ | CBS 120665 | *Nicotiana tabacum* | Iran | MH484736 | MH485100 | MH484918 | MH485009 |
| ***F. udum***^c^ | CBS 177.31 | *Digitaria eriantha* | South Africa | MH484775 | MH485048 | MH484866 | MH484957 |

^a^ *cmdA*: calmodulin; *tub2*: β-tubulin; *rpb2*: RNA polymerase II second largest subunit; *tef1*: translation elongation factor 1-alpha.

^b^ out group for phylogeny analysis to FSSC

^c^ out group for phylogeny analysis to FOSC

**Supplementary Table 3.** *Fusarium* *oxysporum* species complex isolates obtained from orchid hosts and locations in Taiwan were used to conduct multilocus phylogenetic analysis

| **Species** | **Isolate code** | **Source** | **Origin** | **GenBank accession number**^a^ | | | |
| --- | --- | --- | --- | --- | --- | --- | --- |
|  |  |  |  | ***cmdA*** | ***tub2*** | ***rpb2*** | ***tef1*** |
| ***F. contaminatum*** | De18 | *Dendrobium* *nobile* hybrid | Mingjian Township, Nantou County | LC745863 | LC745839 | LC745466 | LC745413 |
|  | De23 | *D. nobile* hybrid | Mingjian Township, Nantou County | LC745864 | LC745840 | LC745467 | LC745414 |
|  | De24 | *D.* *nobile* hybrid | Mingjian Township, Nantou County | LC770340 | LC770332 | LC770366 | LC770348 |
|  | F7 | *Anoectochilus formosanus* | Puli Township, Nantou County | LC745868 | LC745844 | LC745471 | LC745418 |
|  | Le91 | *An. formosanus* | Puli Township, Nantou County | LC745871 | LC745847 | LC745474 | LC745421 |
| ***F. cugenangense*** | FuTn7s | *Phalaenopsis* sp. | Tainan City | LC863983 | LC864397 | LC864435 | LC864394 |
| ***F. curvatum*** | Ca5 | *Cattleya* sp. | Mingjian Township, Nantou County | LC745857 | LC745833 | LC745460 | LC745407 |
|  | CyB04 | *Cymbidium ensifolium* | South District, Taichung City | LC745861 | LC745837 | LC745464 | LC745411 |
|  | CyB05 | *Cy. ensifolium* | South District, Taichung City | LC780600 | LC780602 | LC780604 | LC780501 |
|  | CyB06 | *Cy. ensifolium* | South District, Taichung City | LC745862 | LC745838 | LC745465 | LC745412 |
|  | CyB07 | *Cy. ensifolium* | South District, Taichung City | LC863597 | LC863984 | LC864399 |  |
|  | CyB13 | *Cy. ensifolium* | South District, Taichung City | LC863598 | LC863985 | LC864400 | LC780507 |
|  | CyB14 | *Cy. ensifolium* | Houli District, Taichung City | LC863599 | LC863986 | LC864401 | LC780508 |
|  | CyB24 | *Cy. ensifolium* | Yuchi Township, Nantou County | LC863600 | LC863987 | LC864402 | LC780509 |
|  | CyB26 | *Cy. ensifolium* | Yuchi Township, Nantou County | LC863601 | LC863988 | LC864403 | LC780511 |
|  | CyB31 | *Cy. ensifolium* | Yuchi Township, Nantou County | LC863604 | LC863991 | LC864406 | LC780516 |
|  | CyB33 | *Cy. ensifolium* | Yuchi Township, Nantou County | LC863605 | LC863992 | LC864407 | LC780518 |
|  | CyB34 | *Cy. ensifolium* | Yuchi Township, Nantou County | LC863606 | LC863993 | LC864408 | LC780519 |
|  | CyB35 | *Cy. ensifolium* | Yuchi Township, Nantou County | LC863607 | LC863994 | LC864409 | LC780520 |
|  | CyB43 | *Cy. ensifolium* | Meishan Township, Chiayi County | LC863609 | LC863996 | LC864406 | LC780527 |
|  | CyB55 | *Cy. ensifolium* | Gukeng Township, Yunlin County | LC863610 | LC863997 | LC864407 | LC780532 |
|  | CyB57 | *Cy. ensifolium* | Gukeng Township, Yunlin County | LC863611 | LC863998 | LC864413 | LC780534 |
|  | CyB58 | *Cy. ensifolium* | Gukeng Township, Yunlin County | LC863612 | LC863999 | LC864414 | LC780535 |
|  | CyB61 | *Cy. ensifolium* | Meishan Township, Chiayi County | LC863613 | LC864000 | LC864415 | LC780538 |
|  | CyB63 | *Cy. ensifolium* | Meishan Township, Chiayi County | LC863614 | LC864001 | LC864416 | LC780540 |
|  | CyB67 | *Cy. ensifolium* | Meishan Township, Chiayi County | LC863616 | LC864003 | LC864418 | LC780543 |
|  | De29 | *Dendrobium* sp. | Douliu City, Yunlin County | LC745865 | LC745841 | LC745468 | LC745415 |
|  | De30 | *Dendrobium* sp. | Douliu City, Yunlin County | LC745866 | LC745842 | LC745469 | LC745416 |
|  | Fo-92 | *Cymbidium* sp. | Wufeng District, Taichung City | LC770342 | LC770334 | LC770368 | MN162687 |
|  | FuTn29r | *Phalaenopsis* sp. | Tainan city | LC863982 | LC864398 | LC864436 | LC864395 |
|  | Ha1-1 | *Haraella retrocalla* | South District, Taichung City | LC745870 | LC745846 | LC745473 | LC745420 |
|  | Pa18 | *Paphiopedilum* sp. | Puli Township, Nantou County | LC745873 | LC745849 | LC745476 | LC745423 |
|  | Pa19 | *Paphiopedilum* sp. | Puli Township, Nantou County | LC863620 | LC864004 | LC864419 | LC780544 |
|  | Pa23 | *Pa. callosum* | Puli Township, Nantou County | LC745874 | LC745850 | LC745477 | LC745424 |
|  | Pa24 | *Pa. callosum* | Puli Township, Nantou County | LC770344 | LC770336 | LC770370 | LC770350 |
|  | Pa25 | *Pa. callosum* | Puli Township, Nantou County | LC863621 | LC864005 | LC864420 | LC780545 |
|  | Pa26 | *Pa. callosum* | Puli Township, Nantou County | LC863622 | LC864006 | LC864421 | LC780546 |
|  | Pa27 | *Pa. callosum* | Puli Township, Nantou County | LC863623 | LC864007 | LC864422 | LC780547 |
|  | Pa28 | *Pa. callosum* | Puli Township, Nantou County | LC863624 | LC864008 | LC864423 | LC780548 |
|  | Va3 | *Vanda* *ampullaceum* | Huwei Township, Yunlin County | LC745876 | LC745852 | LC745479 | LC745426 |
| ***F. nirenbergiae*** | Cy41 | *Cy. sinense* | Houli District, Taichung City | LC745860 | LC745836 | LC745463 | LC745410 |
|  | FuC2r | *Phalaenopsis* sp. | Chiayi County | LC863981 | LC864396 | LC864434 | LC864393 |
|  | Ma6 | *Maxillaria tenuifolia* | Houli District, Taichung City | LC770343 | LC770335 | LC770369 | LC770349 |
|  | N8284 | *Phalaenopsis* sp. | **-** | LC864019 | LC864438 | LC864439 | LC864437 |
|  | Pa17 | *Paphiopedilum* sp. | Dacun Township, Changhua County | LC745872 | LC745848 | LC745475 | LC745422 |
|  | Va40-2 | *Arachnis* x *Vanda* | Zhutian Township, Pingtung County | LC770345 | LC770337 | LC770371 | LC770351 |
|  | Va45 | *Arachnis* x *Vanda* | Zhutian Township, Pingtung County | LC745877 | LC745853 | LC745480 | LC745427 |
|  | Va48 | *Arachnis* x *Vanda* | Zhutian Township, Pingtung County | LC745878 | LC745854 | LC745481 | LC745428 |
|  | VaP1 | *Vanilla planifolia* | Puli Township, Nantou County | LC863627 | LC86400 | LC864426 | LC780549 |
|  | VaP2-1 | *Vani. planifolia* | Puli Township, Nantou County | LC863628 | LC864013 | LC864427 | LC780550 |
|  | VaP3-1 | *Vani. planifolia* | Puli Township, Nantou County | LC863633 | LC864017 | LC864432 | LC780551 |
|  | VaP4 | *Vani. planifolia* | Puli Township, Nantou County | LC863634 | LC864018 | LC864433 | LC780552 |
|  | VaP5 | *Vani. planifolia* | Puli Township, Nantou County | LC745879 | LC745855 | LC745482 | LC745429 |
|  | VaP6 | *Vani. planifolia* | Puli Township, Nantou County | LC745880 | LC745856 | LC745483 | LC745430 |
|  | VaP12 | *Vani. planifolia* | Puli Township, Nantou County | LC863625 | LC864010 | LC864424 | LC780553 |
|  | VaP14 | *Vani. planifolia* | Puli Township, Nantou County | LC863626 | LC864011 | LC864425 | LC780554 |
|  | VaP15 | *Vani. planifolia* | Puli Township, Nantou County | LC770346 | LC770338 | LC770372 | LC770352 |
|  | VaP17-1 | *Vani. planifolia* | Puli Township, Nantou County | LC770347 | LC770339 | LC770373 | LC770353 |
|  | VaP20 | *Vani. planifolia* | Puli Township, Nantou County | LC863629 | LC864012 | LC864428 | LC780555 |
|  | VaP22 | *Vani. planifolia* | Puli Township, Nantou County | LC863630 | LC864014 | LC864429 | LC780556 |
|  | VaP23 | *Vani. planifolia* | Puli Township, Nantou County | LC863631 | LC864015 | LC864430 | LC780557 |
|  | VaP24 | *Vani. planifolia* | Puli Township, Nantou County | LC863632 | LC864016 | LC864431 | LC780558 |
| ***F. odoratissimum*** | F1 | *An. formosanus* | Puli Township, Nantou County | LC745867 | LC745843 | LC745470 | LC745417 |
|  | FL1409 | *An. formosanus* | Puli Township, Nantou County | LC745869 | LC745845 | LC745472 | LC745419 |
|  | Cy25 | *Cy. sinense* | Houli District, Taichung City | LC745858 | LC745834 | LC745461 | LC745408 |
|  | Cy28 | *Cy. sinense* | Houli District, Taichung City | LC745859 | LC745835 | LC745462 | LC745409 |
|  | Cy32 | *Cy. sinense* | Houli District, Taichung City | LC780599 | LC780601 | LC780603 | LC780498 |
|  | CyB27 | *Cy. ensifolium* | Yuchi Township, Nantou County | LC863602 | LC863989 | LC863989 | LC780512 |
|  | CyB30 | *Cy. ensifolium* | Yuchi Township, Nantou County | LC863603 | LC863990 | LC863990 | LC780515 |
|  | CyB41 | *Cy. ensifolium* | Yuchi Township, Nantou County | LC863608 | LC863995 | LC863995 | LC780525 |
|  | CyB65 | *Cy. ensifolium* | Meishan Township, Chiayi County | LC863615 | LC864002 | LC864002 | LC780542 |
|  | Fo-51 | *Cymbidium* sp. | Nantou County | LC770341 | LC770333 | LC770367 | MN162686 |
|  | Pa29 | *Pa callosum* | Puli Township, Nantou County | LC745875 | LC745851 | LC745478 | LC745425 |

^a^ *cmdA*: calmodulin; *tub2*: β-tubulin; *rpb2*: RNA polymerase II second largest subunit; *tef1*: translation elongation factor 1-alpha.
